# Detecting changes in population trends in infection surveillance using community SARS-CoV-2 prevalence as an exemplar

**DOI:** 10.1093/aje/kwae091

**Published:** 2024-05-29

**Authors:** Emma Pritchard, Karina-Doris Vihta, David W. Eyre, Susan Hopkins, Tim EA Peto, Philippa C. Matthews, Nicole Stoesser, Ruth Studley, Emma Rourke, Ian Diamond, Koen B. Pouwels, Ann Sarah Walker, COVID-19 Infection Survey Team

**Affiliations:** 1 Nuffield Department of Medicine, University of Oxford, Oxford, UK; 2 The National Institute for Health Research Health Protection Research Unit in Healthcare Associated Infections and Antimicrobial Resistance at the University of Oxford, Oxford, UK; 3 Department of Engineering, University of Oxford, Oxford, UK; 4 The National Institute for Health Research Oxford Biomedical Research Centre, University of Oxford, Oxford, UK; 5 Big Data Institute, Nuffield Department of Population Health, University of Oxford, Oxford, UK; 6 Healthcare-Associated Infection and Antimicrobial Resistance Division, Public Health England, London, UK; 7 National Institute for Health Research, Health Protection Research Unit in Healthcare Associated Infections and Antimicrobial Resistance, Imperial College London, London, UK; 8 Department of Infectious Diseases and Microbiology, Oxford University Hospitals NHS Foundation Trust, John Radcliffe Hospital, Oxford, UK; 9 Francis Crick Institute, London, UK; 10 Division of Infection and Immunity, University College London, London UK; 11 Department of Infection, University College London Hospitals, London, UK; 12 Office for National Statistics, Newport, UK; 13 Health Economics Research Centre, Nuffield Department of Population Health, University of Oxford, Oxford, UK

## Abstract

Detecting and quantifying changes in growth rates of infectious diseases is vital to informing public health strategy and can inform policymakers’ rationale for implementing or continuing interventions aimed at reducing impact. Substantial changes in SARS-CoV-2 prevalence with emergence of variants provides opportunity to investigate different methods to do this. We included PCR results from all participants in the UK’s COVID-19 Infection Survey between August 2020-June 2022. Change-points for growth rates were identified using iterative sequential regression (ISR) and second derivatives of generalised additive models (GAMs). Consistency between methods and timeliness of detection were compared. Of 8,799,079 visits, 147,278 (1.7%) were PCR-positive. Change-points associated with emergence of major variants were estimated to occur a median 4 days earlier (IQR 0-8) in GAMs versus ISR. When estimating recent change-points using successive data periods, four change-points (4/96) identified by GAMs were not found when adding later data or by ISR. Change-points were detected 3-5 weeks after they occurred in both methods but could be detected earlier within specific subgroups. Change-points in growth rates of SARS-CoV-2 can be detected in near real-time using ISR and second derivatives of GAMs. To increase certainty about changes in epidemic trajectories both methods could be run in parallel.

Infectious disease surveillance has two broad goals; identifying outbreaks which lead to sudden changes in incidence/prevalence, and detecting emergence of more virulent/resistant strains. Reasons for changes in infectious disease trends vary, for example: changing population susceptibility causing increasing group A streptococcal infections;^1^ emerging antimicrobial resistant strains e.g. ribotype-027 *Clostridium difficile* or gram-negative pathogens carrying extended-spectrum beta-lactamases;^2^ and mutations affecting transmissibility in COVID-19.^3^ While laboratory sequencing methods can accurately identify variants,^4^ limited sampling and resources mean retrospective statistical models are often more practical for monitoring infectious diseases, particularly with increasing availability of linked electronic health records.

While change-point detection methods are numerous, many are sub-optimal for use on infectious disease time-series in near real-time. Statistical methods involve locating points in a time-series where some property of the data (e.g. distribution, scale) changes.^5^ Given the epidemiological drivers above, identifying points where the rate of change in the trend is increasing/decreasing are most useful in infectious disease surveillance. Many statistical methods however identify step changes, i.e. changes in mean levels in a time-series, rather than the more gradual trend changes^6^ characteristic of changing infectious disease epidemiology. Other methods require pre-specifying the number of change-points, and many are computationally expensive therefore not practical for near real-time use. During the COVID-19 pandemic, while studies used change-point analysis to retrospectively assess the impact of interventions e.g. lockdowns and gatherings,^7–9^ change-point detection methods for near real-time use have been less commonly assessed.^10^ Two methods considering more gradual changes and finding change-points in trends are iterative sequential regression^11,12^ (ISR) and second derivatives of generalised additive models (GAMs).^13^ While both have been evaluated separately,^11,14^ to our knowledge, they have never been directly compared. ISR provides a clear statistical assessment of when rates change and estimates constant growth rates between change-points, potentially maximizing power when this is close to true underlying trends. However, it considers data sequentially, fixing change-points as it iterates, thus not necessarily optimising overall fit. Second derivatives of GAMs have been used to identify periods of change,^14,15^ and quantify change-points.^13^ Their flexibility allows estimates to closely reflect reality, but to what extent smoothing through penalized splines reduces the ability to detect change-points in near real-time is unclear.

We aimed to compare the performance of GAMs and ISR for change-point detection for infectious disease surveillance, both retrospectively and in near real-time, using COVID-19 as an exemplar for surveillance more generally, e.g. using linked electronic health records. Rapid changes in SARS-CoV-2 prevalence coupled with multiple emerging variants over the COVID-19 pandemic provides an ideal opportunity to test these methods in real-world data which is more complex than simulations. We compared the consistency and timeliness of detection between ISR and second derivatives of GAMs for identifying changes in growth rates of SARS-CoV-2 positivity over time using the UK’s Office for National Statistics (ONS) COVID-19 Infection Survey. We assessed whether earlier detection was possible considering positivity separately by age group or by available proxies for viral variant.

## Methods

### Study design

The ONS COVID-19 Infection Survey was a large household survey with longitudinal follow-up (ISRCTN21086382). Private households were continuously selected randomly from address lists (non-response in^16^) and previous ONS surveys to provide a broadly representative sample across the UK^17^ (Supplementary Tables 3-6 in^18^). Following verbal consent, study workers visited each household to take written informed consent for individuals ≥2 years (from parents/carers for those 2–15 years; those 10–15 years also provided written assent). The study received ethical approval from the South Central Berkshire B Research Ethics Committee (20/SC/0195). At the first visit, participants were asked for consent for optional follow-up visits weekly for the next month, then monthly thereafter (>98.5% providing such consent). At each visit, participants provided a nose and throat self-swab and completed questionnaires (https://www.ndm.ox.ac.uk/covid-19/covid-19-infection-survey/case-record-forms).

### Study Population

Analysis included all visits with positive or negative swabs from 1^st^ August 2020-30th June 2022 (n=225,348 (2%) visits with void/missing results excluded).

### Statistical Analyses

Our outcome measure was the proportion of visits with PCR-positive SARS-CoV-2 tests. We compared two methods for detecting changes in trend over time: ISR^11^ and second derivatives of GAMs.^13^ All models were run separately for 12 geographical regions (9 English regions and 3 devolved administrations: Wales, Scotland, Northern Ireland) due to positivity trend differences. ISR estimates change-points in a single time-series; separate GAMs by region differed only slightly from GAMS including region-time interactions, and reduced computational time (Supplementary Figure S1).

ISR, using a negative-binomial distribution with log link allowing overdispersion, initially fitted a log-linear trend within the first month’s data to 1st September 2020. Three days’ data were sequentially added to the time-series, fixing change-points if a new trend reduced the AIC by ≥6.635 (critical value at p=0.01, to reduce the impact of false-positives). If a change-point was fixed, a new change-point was not considered in the subsequent seven days. Change-points, and dates change-points were permanently fixed into the model (“detection date”), were extracted from fitted models (**Supplementary Methods**).

GAMs, using a negative-binomial distribution with log link, included a single explanatory variable of time in days since 1^st^ August 2020 and modelled using thin plate splines.^19^ The number of basis functions, *k*, determining smoothness, was selected from 25, 50, 75, 100 as the lowest value with predicted positivity within ±0.25% (absolute scale) versus *k*=100, optimising computational time, without large increases in the effective degrees of freedom^20^ (Supplementary Figure S2). Splines were penalised based on the third derivative as the second derivative was the measure of interest.

Derivatives were estimated for smooth functions using posterior simulation with a Metropolis-Hastings sampler (as implemented in the gam.mh function from the *mgcv* R package)^21,22^ as standard Gaussian approximation will be poor in low positivity periods (details in **Supplementary Methods**). Code was adapted from the *derivatives* function in the R *gratia* package, which currently can only obtain derivatives on the linear predictor scale.^23^ Change-points were defined at the first day zero was excluded from the 95% credible interval of the second derivative, corresponding to 97.5% probability of change, from 1^st^ September 2020 onwards. Positivity trends over the full time-series were compared between ISR and GAMs. Change-points were classified as found by both methods if within ±7 days, an arbitrary but pragmatic window based on the distribution of time between change-points identified by both methods and timeliness of public health responses (**Supplementary methods; Figure S3**). Change-points corresponding to Alpha, Delta, BA.1, and BA.2 variant emergence were compared between methods.

As ISR fixes change-points once found and adds data progressively, it does not need to be run on segments of data sequentially to assess near real-time detection. In near real-time, one could run ISR from the latest detected change-point onwards to decrease fitting time, albeit change-points may differ slightly versus models incorporating the full time-series as previous data can impact AIC. To compare near real-time detection between methods, GAMs were run successively adopting a sliding-window approach. Sliding-window length was determined by running GAMs on shorter periods (16, 24, and 32-weeks) and assessing whether similar change-points were found in the final 8-weeks as most recent changes are of most interest in near real-time. Starting from 1st October 2020 (including data from 1st August 2020), seven-day increments of data were added until the sliding-window length was reached, from which seven-days of data were removed from the start of the time-series each time seven-days were added on. We selected *k* as before for sliding-window length, scaling *k* down proportionally for the shorter time-series. We assessed whether all change-points identified in the last 8-weeks of each model were detected within ±7 days in five subsequent models and/or by ISR. Due to long runtimes (~12-36 hours per region including derivatives estimation), we compared GAM detection dates for the largest (London) and smallest (Northern Ireland) regions. For each change-point identified in the GAM including data from the full time-series, we defined the “detection date” as the last date included in the earliest successive GAM which also confirmed the change-point within ±7 days. We compared change-points identified in the last 4-weeks of the weekly successive GAMs with change-points found in the full time-series GAM to quantify the false positivity and negativity rates for successive GAMs to identify recent change-points.

Using the second derivative of GAMs risks potentially missing change-points if positivity decreases and increases at the same rate over a short time period. While the second derivative will be significantly different from zero, new change-points will not be found when positivity changes direction as the second derivative may not cross zero. We summarised the number and position of additional change-points added if placed where, over periods of second derivative significance, the first derivative changed from significantly positive to negative, or visa-versa.

### Sensitivity Analysis

To assess whether earlier detection of change-points was possible by focusing on high-risk population subgroups, change-points estimated from separate ISR and GAMs in those aged 2 years (y)-school year (sy) 11 (~ aged 16y), 12sy–49y, and 50y+, were compared with combined all-age estimates. We considered separate analysis by PCR gene positivity as a proxy for SARS-CoV-2 variant; Delta and BA.2 being spike (S) gene target positive (SGTP), whereas Alpha and BA.1 had S-gene target failure (SGTF). Models were run separately with SGTP and SGTF positivity as outcomes, with all other positives (including those positive on only the N gene or ORF1ab) in the negative comparator group, comparing change-points to the “all positives” model.

All analysis was conducted in R version 4.0.2. Key analysis code is available at https://github.com/EmmaPritchard.

## Results

**Figure 1:**
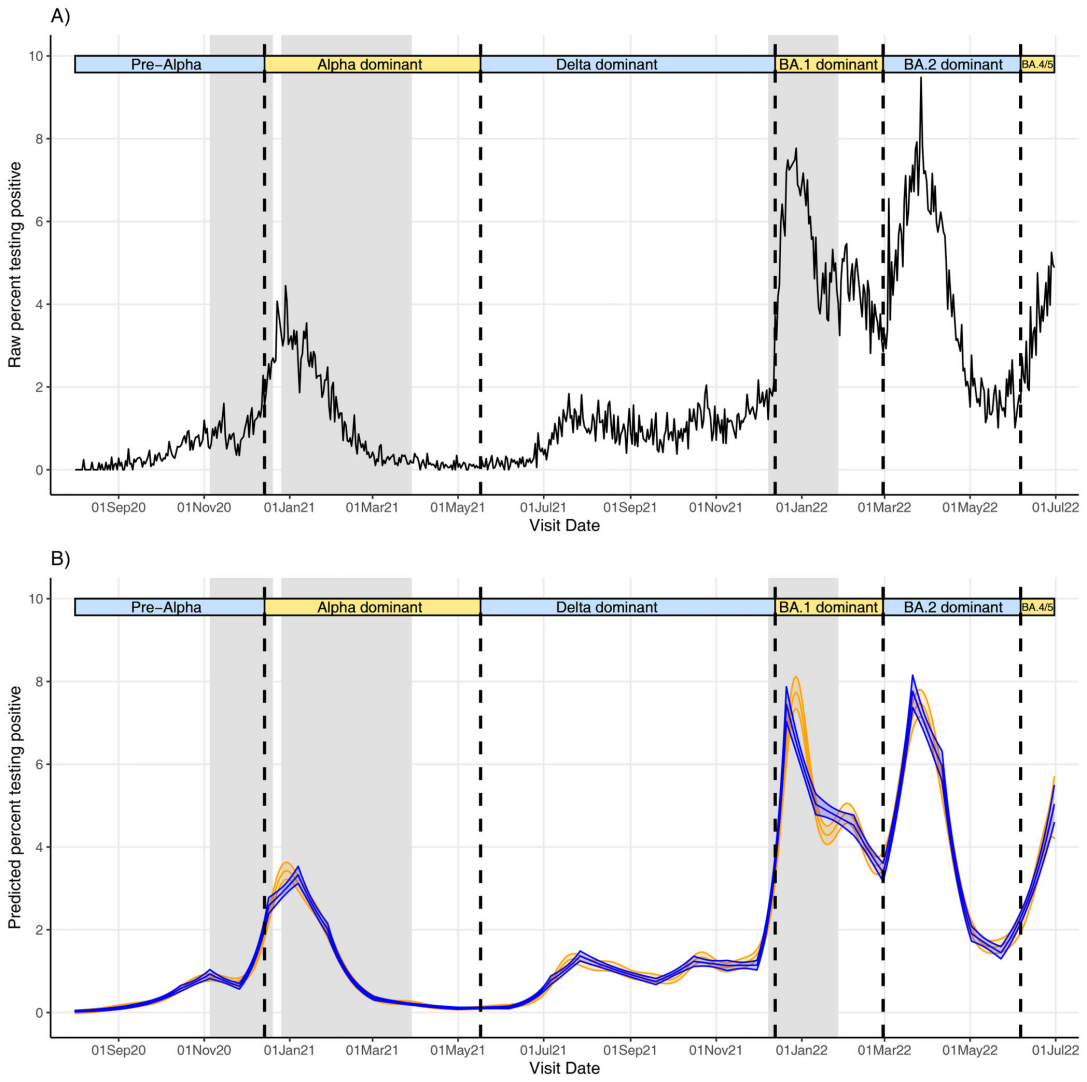
**Raw percentage testing positive (A) and predicted percentage of visits testing positive (B) for SARS-CoV-2 from ISR (blue) and GAMs (orange) between August 2020-June 2022 for London only.** Note: Vertical dashed lines indicate periods when new variants became dominant, defined as >50% of positive swabs with cycle threshold (Ct)<30 being S-gene target positive (ORF1ab+N+S, ORF1ab+S, N+S gene positivity) in the Covid-19 Infection Survey for the pre-Alpha period (01 August 2020 - 13 December 2020), the Delta variant (17 May 2021 – 12 December 2021), and the Omicron BA.2 variant (28 February 2022 – 5 June 2022), and >50% Ct<30 S-gene target negative (ORF1ab+N gene positivity) for the Alpha variant (14 December 2020 – 16 May 2021), Omicron BA.1 variant (13 December 2021 – 27 February 2022), and Omicron BA4/BA.5 (6 June 2022 onwards). Gray shaded indicate periods where stay/work from home laws were enforced, although specific restrictions varied across the time series.

From 1^st^ August 2020-30th June 2022, 8,799,079 visits from 533,157 participants in 266,400 households returned 147,278 (1.7%) SARS-CoV-2 positive swabs (characteristics in Supplementary Table S1). From August-November 2020 (pre-Alpha), positivity rose to ~1%, before increasing to ~2% in January 2021 (Alpha; Figure 1A; Supplementary Figure S4). Positivity decreased until June 2021 before increasing to ~1-2% in July-December 2021 (Delta). Positivity rose sharply to ~6% from December 2021 (BA.1), decreasing to ~3.5% by February 2022, before increasing to ~7.5% by mid-March (BA.2). Rises with BA.4/BA.5 began June 2022. During the pre-Alpha period, 10% of strong positives (Ct<30) had SGTF, versus 79%, 1%, 84%, and 9% in Alpha, Delta, BA.1, and BA.2-dominant periods (Supplementary Table S2). Positivity varied by region, particularly between Northern/Southern English regions e.g. higher positivity pre-Alpha in Yorkshire, versus London (Supplementary Figure S4B).

### Detecting changes in growth rates using ISR and GAMs

**Figure 2:**
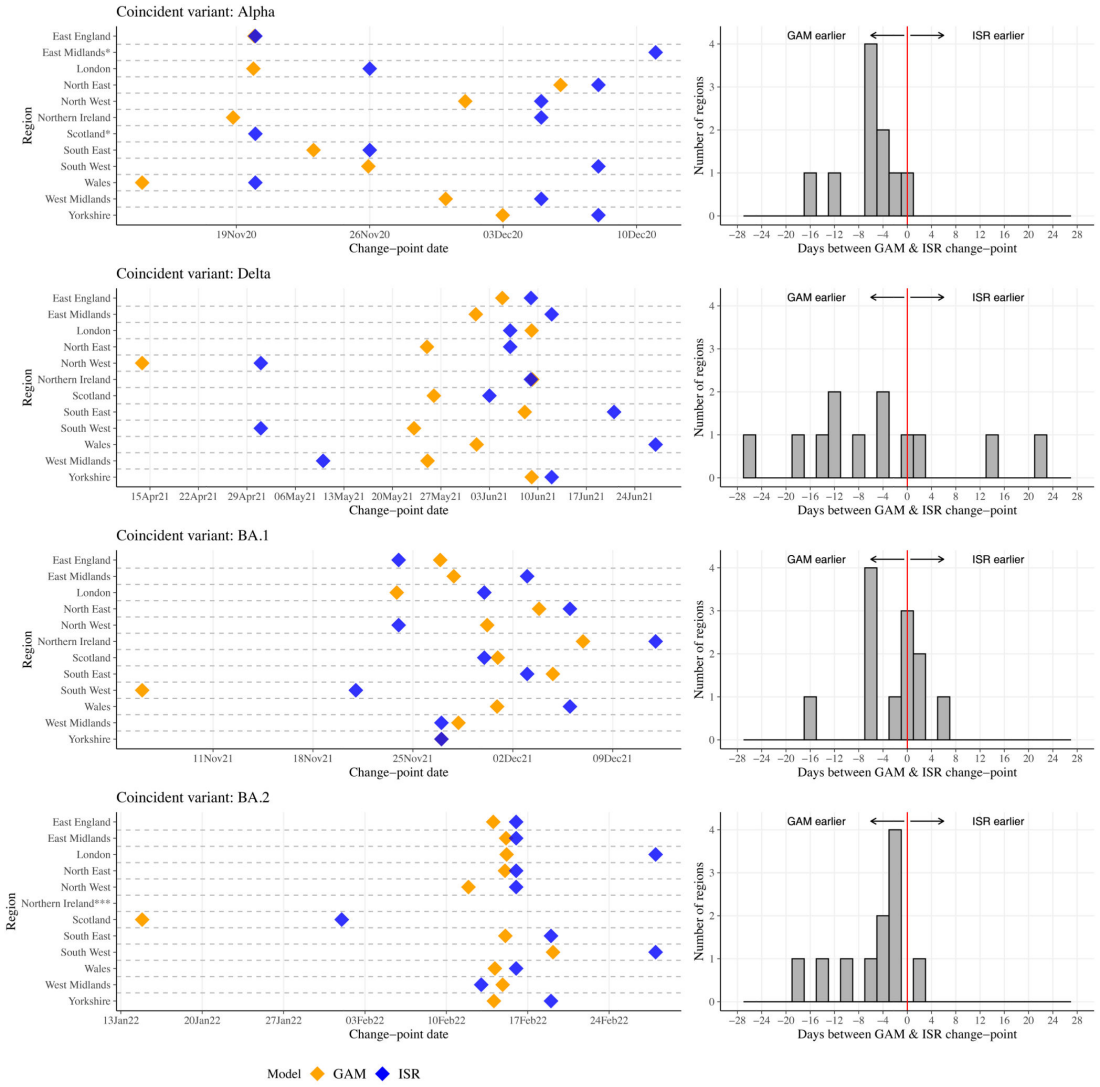
**Change-points corresponding to emergence of three key SARS-CoV-2 variants found by iterative sequential regression (ISR) and second derivatives of generalised additive models (GAMs). ISR and GAMs were run separately for each of the 12 geographical regions presented and all run on the full time-series between August 2020-June 2022.** Note: *No change-point coincident with variant using GAMs; **No change-point coincident with variant using ISR; ***No change-point coincident with variant for GAMs and ISR. Exact dates of change-points are in Table S3.

We compared change-points detected by the two methods, first considering emergence of dominant SARS-CoV-2 variants. ISR and GAMs made similar predictions of changing positivity trends across geographical regions over the study period (Figure 1B, Supplementary Figure S5). In London, change-points corresponding to the emergence of Alpha, Delta, BA.1, and BA.2 occurred on 26 November 2020, 6 June 2021, 30 November 2021, and 28 February 2022 using ISR (Figure 2, Supplementary Table S3), and 6 days earlier, 3 days later, 6 and 13 days earlier, respectively, using GAM derivatives. Across all regions, change-points for the four variants occurred a median 4 days earlier (IQR 0-8) [range 22 days later-26 days earlier] in GAMs versus ISR. 33/48 (69%) change-points occurred earlier using GAMs. No change-point was detected for Alpha in the East Midlands or Scotland using GAMs, but were detected using ISR.

Both methods identified other change-points aside from trend increases resulting from emerging variants, described for London in Table 1. 63% (12/19) of all change-points in London identified in GAMs were identified using ISR within ±7 days. 57% (12/21) of all change-points in London identified by ISR were identified by GAMs within ±7 days. Inconsistent change-points between methods generally reflected small fluctuations when positivity was low. 33% of change-points representing increasing positivity from GAMs and ISR were followed by relative percentage positivity increases >150% (**Supplementary Results**, Supplementary Figure S6).

### Detecting change-points in ‘near real-time’

While retrospectively detecting change-points can quantify how epidemic growth has varied, ideally change-points would be detected in near real-time to inform measures intended to control growth. Comparing GAMs run on double (16-weeks), triple (24-weeks), or quadruple (32-weeks) an arbitrary but realistic 8-week period of interest showed 32-weeks data was the minimum that avoided missing over half the change-points in the full time-series (Supplementary Table S4, **Supplementary Results**).

**Figure 3:**
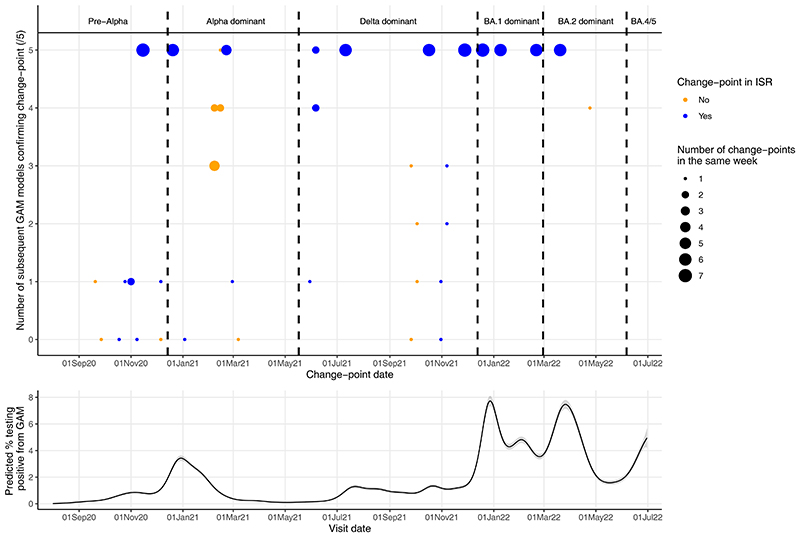
**Change-points found by generalised additive models (GAMs) run successively over 32-week periods from September 2020-June 2022 summarised by the number of successive GAMs each change-point was confirmed by (size of circle; zero to five) and whether the change-points were identified by iterative sequential regression (ISR; colour of circle) (top panel). Predicted positivity from final GAM for reference (bottom panel). Results are for London only.** Note: Change-points in the same week (starting Monday) found in the same number of subsequent models were grouped together (indicated by size of circle). Points are blue if at least one change-point in that week was also found by ISR, and orange is no change-points in that week were found by ISR.

Running GAMs successively adding new data for London every week from 1^st^ October 2020-30th June 2022, 96 change-points were found in the final 8-weeks across all GAMs (Figure 3). Most (64/96: 67%) change-points were identified by five successive GAMs. Eight (8%) change-points were not identified in any of the five subsequent GAMs, but four of these were identified by ISR. Overall, 77% (74/96) of change-points in the last 8-weeks of successive GAMs were identified by ISR, and 23% (22/96) were never identified by ISR. Results were similar for Northern Ireland (**Supplementary Results**; Supplementary Figure S7). Change-points in the final 4-weeks of successive GAMs found 62% (8/13) of change-points in the full time-series GAM, including for all major variant increases (Supplementary Table S5, **Supplementary Results**).

**Figure 4:**
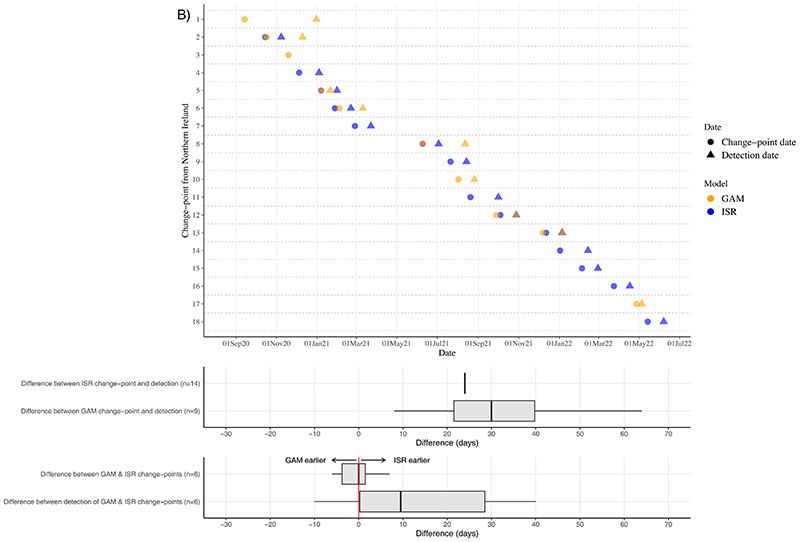
**Dates of change-points (circles) and the date at which the change-point would have first been detected in near to real-time (detection dates; triangles) from generalised additive models (GAMS; orange) and iterative sequential regression (ISR; blue), separately for London (A) and Northern Ireland (B), between September 2020-June 2022. Box plots summarise the median and IQR number of days between ISR and GAM change-points and their detection dates.**

Using the final date of the first successive GAM to estimate when change-points in the full time-series GAM would have been detected in near real-time, for London, change-points were detected a median 21 (IQR 17-26; range 10-128) days later (Figure 4; Supplementary Table S6). ISR generally fixed change-points into the model (based on lower AIC vs linear trend) 24 days after the change. When identified by both GAMs and ISR, successive GAMs detected change-points a median 4 (IQR 10 days earlier, 1 day later; range 17 days earlier-35 days later) days earlier. Four change-points identified in the final GAM for London were not identified in any successive GAMs, hence detection dates could not be determined.

When considering change-points for Northern Ireland (around a fifth of the visits from London), while ISR still detected change-points ~24 days after the change occurred, GAMs detected changes median 30 (IQR: 24-54; range: 8-108) days later (Figure 4; Supplementary Table S6). When identified by both ISR and GAMs, in contrast to London, ISR detected change-points a median 10 (IQR: 0,32) days earlier.

### Incorporating change-points based on the first derivative

Change-points for BA.4/BA.5 were found for all regions using ISR, but were not found using GAMs for 9/12 regions (Supplementary Table S7). The BA.4/BA.5 growth rate was similar to BA.2 decline so, while the second derivative was significantly different from zero, new change-points were not established. Adding in additional change-points where the first derivative switched signs, GAMs found change-points for BA.4/BA.5 in all regions (Supplementary Figure S8). See **Supplementary Results** for further details on additional change-points established by first derivatives.

### Estimating change-points in target subgroups

Analogous to “sentinel surveillance”, we assessed whether change-points could be established earlier by modelling population subgroups, here age. In our dataset, as others,^17^ large rises in positivity associated with Alpha emergence occurred earlier in those 2y-11sy, with steeper increases in positivity in late-August 2021 (Delta) and late-January 2022 (BA.1) versus older age-groups (Supplementary Figure S9, S10).

**Figure 5:**
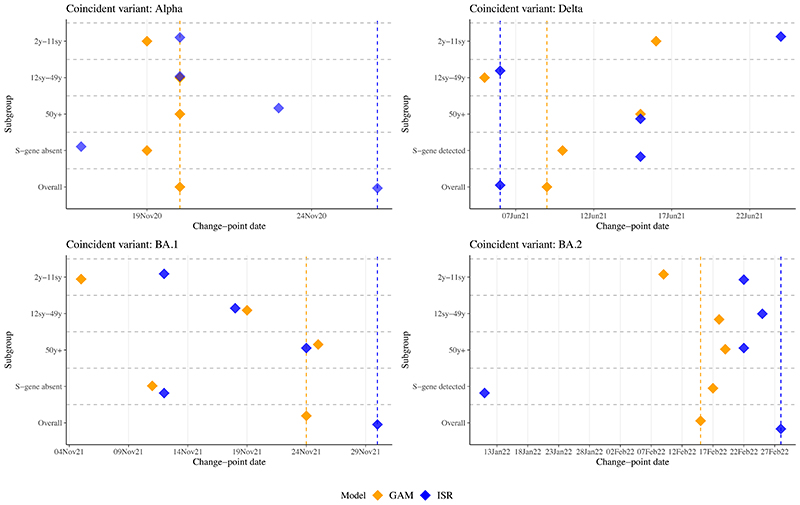
**Change-points from generalised additive models (GAMs; orange) and iterative sequential regression (ISR; blue) run separately by age, separately by S gene detection, and overall in London. All models included data from the full time-series (between August 2020-June 2022).** Note: Vertical dashed lines show position of change-points in overall GAM and ISR. Change-point dates are provided in Table S8.

Little difference was seen across age groups for GAM change-points associated with Alpha (Figure 5). For Delta, change-points occurred earliest in the overall model and those 12sy-49y, and latest in those 2y-11sy. Rises in BA.1 occurred 18 days earlier in the youngest age group versus all ages using ISR, and 19 days earlier using GAMs. Rises in BA.2 were found earliest in those 2y-11sy using GAMs (9 February 2022).

### Estimating change-points by outcome type

Analogous to surveillance of different infection types, e.g. resistant vs susceptible *Staphylococcus aureus*, we considered whether change-points could be established earlier by modelling PCR S-gene positivity as a proxy for SARS-CoV-2 variant. There were distinct trend differences between SGTF and SGTP positivity over time (Supplementary Figure S11, S12) and GAM and ISR predictions for London closely followed these (Supplementary Figure S13).

For London, change-points associated with Alpha emergence occurred one and nine days earlier using SGTF versus all positives for GAMs and ISR, respectively (Figure 5). Change-points for Delta occurred and were detected nine days later using SGTP versus all positives using ISR, and occurred one day later using GAMs. Change-points for BA.1 occurred on 11 and 12 November 2021 using GAMs and ISR for SGTF, with all-positive change-points on 24 and 30 November 2021, respectively, a 15-day earlier detection for ISR. For SGTP, ISR estimated a change-point for BA.2 on 11 January 2022 (detected 4 February 2022) and did not find a change-point for all-positives until 48 days later.

## Discussion

Here, we compared two methods for detecting changes in growth rates in surveillance data, using SARS-CoV-2 as an exemplar. Both methods detected trend increases and decreases associated with Alpha, Delta, BA.1, and BA.2 variants, and other smaller growth rate fluctuations, at similar dates. Considering near real-time analysis, most recent change-points detected using GAMs were found in successive GAMs including five subsequent weeks’ data and using ISR, demonstrating consistency between GAM model runs and between methods. However, GAMs needed at least 4 times the duration of data over which there was interest in identifying change-points to provide stable estimates. Change-points were, on average, detected slightly earlier using GAMs versus ISR considering larger geographical regions, but this was not consistent across different sized regions or subgroups. Considering positivity trends separately in different age subgroups allowed earlier BA.1 detection using data from children alone versus all positives and separately for SGTP of BA.2.

Supporting using these methods for sentinel surveillance, we found change-points could be detected earlier through modelling age groups separately, often, but not always, in those 2y-11sy. This is likely due to faster SARS-CoV-2 transmission at younger ages, specifically during Omicron BA.1 emergence, driven by higher contact levels through school attendance while other guidance (e.g. working from home^24^) slowed transmission between adults until infections were later transmitted onwards from younger to older individuals. While studies have found no evidence of increased transmission on school premises,^25–28^ increased person-to-person contact associated with attending school (e.g. public transport, gatherings at school pick-up/drop-off) measurably impact the reproduction number.^29^ Rising SARS-CoV-2 positivity in younger individuals may therefore be a useful early warning signal for rises in older individuals, where hospitalisation risk and mortality are higher,^30^ although trend changes were not consistently identified earlier in younger age groups. Implementing surveillance systems separately by subgroups may be an efficient way to detect changes earlier more generally.

We found change-points were generally estimated to occur slightly earlier when considering positivity split by S-gene detection. This was particularly useful when BA.2 emerged, as BA.1 declines concealed fast BA.2 growth when combining all positives. More broadly, surveillance of pathogens with different susceptibilities could allow similar shifts in underlying variants to be elucidated.^31^

The methods have much wider applicability to infection surveillance, but SARS-CoV-2 provided an ideal opportunity to test them due to rapid changes in positivity and emerging variants with different epidemiology. Change-points estimated for Alpha using ISR and GAMs were generally consistent with changing UK public health policy. The first Alpha sequence came from a sample on 20 September 2020, but it was not widely recognized until December after its rapid growth throughout November,^32,33^ with regional lockdowns implemented on 23rd December 2020.^34^ By then, change-points had been detected by ISR in four geographical regions, including London and South East England where Alpha rose earliest and fastest. In contrast, Delta was named a variant of concern on 6 May 2021,^35^ approximately a month earlier than change-points occurred in most regions in our analysis, reflecting its earlier identification through rapid increases in infections in India. The appropriate length of time between changes occurring and change-point detection thus depends on the surrounding context. The average 3-week lag observed here may not be generalisable under large-scale specific testing, but may be relevant if surveillance is reliant on passive data. The infection/disease being monitored could also influence the relevance of this lag. Fundamentally ISR and GAMs identify when infection epidemiology changes, which may be independent of or coincident with recognition of new variants with different transmission potential, virulence, or resistance through genetic sequencing or changes in epidemiology in other countries. Whilst our methods could be applied to the proportion of genetic sequences which are a specific variant, to date this has generally shown log-linear growth for SARS-CoV-2,^36^ without change-points before a new variant becomes the majority sequence.

Real-time surveillance is mostly concerned with recent data, where uncertainty is greatest. Using ISR, most change-points were detected slightly later – a limitation of ISR requiring a minimum number of days between the current and last identified change-point. While GAMs detected some change-points earlier, they also found a small number of change-points during the last 7-days of successive model runs which were not confirmed when adding a further 7-days’ data. The increased flexibility afforded through GAMs may therefore cause false-positives at data boundaries. Most change-points found in the last 4-weeks of successive GAMs were confirmed by the full time-series GAMs, albeit sometimes later due to reduced power over shorter timeframes. Some change-points near the end of successive model runs were not found in the full time-series GAM, possibly due to the full time-series GAMs over smoothing across periods of variation. Requiring at least 7-days of data after change-points, or confirmation in two successive models, would increase certainty. Change-points were found slightly later in GAMs versus ISR on smaller datasets, likely due to ISR fixing one parameter at a time whereas GAMs optimise over the entire time-series, hence being more influenced by sample size. Overall, this illustrates the inherent trade-offs between the two methods; ISR’s forcing of log-linear trends between change-points will identify change-points more efficiently when this is close to the truth, but will be inefficient if trends are volatile.

While most change-points found in this study were related to increases and decreases in major variants, we also identified other fluctuations. Most of these other change-points were associated with large relative percentage changes in positivity over 4-weeks, with most smaller relative changes indicating growth/decline slowing down or flattening off so still epidemiologically important. Some change-points identified by GAMs were significant for short durations e.g. one day, and of small magnitude in the second derivative. Whilst statistically significant, these changes may not be meaningful, with policy decisions more likely made on larger changes in growth/decay. For both GAMs and ISR, we would recommend interpreting change-points in the context of current underlying prevalence. Further, using second derivatives of GAMs, change-points for BA.4/BA.5 were mostly not found by the 30th June 2022 but could be established when considering additional change-points based on the first derivative swapping sign. Considering changes in the first derivative may be important to avoid missing change-points moving forward. As regards methods, estimating derivatives using a Metropolis-Hastings or similar sampler is recommended during low prevalence periods.

In our exemplar, demonstrating that relevant change-points can be detected in a randomly sampled community population is useful for future SARS-CoV-2 surveillance, as this could trigger targeted testing in different regions and/or age groups to help control spread and identify new variants,^37,38^ ultimately aiming to reduce cases/hospitalisations. The large sample size allowed power to detect change-points, despite relatively low positivity rates, enabling us to compare the two methods. Whilst SARS-CoV-2 is a respiratory virus, the methods apply more broadly to different infection surveillance data streams.

Study limitations include comparing the two methods on a single dataset, albeit including multiple change-points of different magnitudes. While these methods have been evaluated independently on other datasets,^11^ further comparisons in other settings may be useful. Comparing methods on complex real-world data is practically useful, but future simulation studies could systematically evaluate statistical properties of these methods against a known “gold standard”, albeit likely based on simpler underlying trends. We matched change-points within ±7-days between methods which may have led to a small amount of misclassification. The amount of data required to detect change-points will depend on the specific outcome, and speed of underlying changes, which will differ between respiratory pathogens (e.g. SARS-CoV-2) and antimicrobial resistance determinants, for example. While ISR and second derivatives of GAMs are two options, other change-point detection methods may also be suitable.

In summary, ISR and second derivatives of GAMs could potentially detect changes in trend in multiple different types of infections in near real-time surveillance, including SARS-CoV-2, but more widely including hospital-acquired infections and antimicrobial-resistant pathogens. While both methods gave a generally consistent pattern, some known changes in the epidemiology of SARS-CoV-2 caused by different variants emerging were identified earlier by GAMs than by ISR and vice-versa, therefore running both methods in parallel would be ideal.

## Supplementary Material

Supplementry

## Figures and Tables

**Table 1:** All changepoints found by iterative sequential regression (ISR) and second derivatives of generalised additive models (GAM) between September 2020-June 2022 for London, UK.

GAM change-point date	ISR change-point date	Description of trend	Days between ISR and GAM change-point
26.09.2020	-	Faster growth	-
-	15.10.2020	Faster growth	-
02.11.2020	05.11.2020	Increase to decrease	-3
20.11.2020	26.11.2020	Decrease to increase (rise in Alpha)	-6
19.12.2020	17.12.2020	Slower growth (slowing down of Alpha)	2
-	07.01.2021	Increase to decrease	-
23.01.2021	28.01.2021	Faster decline	-5
05.02.2021	-	Slower decline	-
-	02.03.2021	Slower decline	-
-	01.05.2021	Decrease to increase	-
09.06.2021	06.06.2021	Faster growth (rise of Delta)	3
12.07.2021	06.07.2021	Slower growth	6
-	27.07.2021	Increase to decrease	-
25.09.2021	19.09.2021	Decrease to increase	6
15.10.2021	16.10.2021	Increase to decrease	-1
01.11.2021	-	Decrease to increase	-
-	09.11.2021	Decrease to increase	-
24.11.2021	30.11.2021	Faster growth (rise of BA.1)	-6
20.12.2021	21.12.2021	Increase to decrease (decline of BA.1)	-1
06.01.2022	11.01.2022	Slower decline	-5
29.01.2022	-	Slower decline	-
-	07.02.2022	Decrease to increase	-
15.02.2022	-	Faster growth (rise of BA.2)	-
-	28.02.2022	Decrease to increase (rise of BA.2)	-
16.03.2022	21.03.2022	Increase to decrease (decline of BA.2)	-5
-	11.04.2022	Faster decline	-
19.04.2022	-	Faster decline	-
-	02.05.2022	Slower decline	-
26.05.2022	23.05.2022	Decrease to increase (rise of BA.4/BA.5)	3

Note: Change-points were classified as found by both models if within ±7 days.

* Negative numbers indicate earlier occurrence of change-points using GAMs, compared with ISR.

## Data Availability

De-identified study data are available for access by accredited researchers in the ONS Secure Research Service (SRS) for accredited research purposes under part 5, chapter 5 of the Digital Economy Act 2017. For further information about accreditation, contact research.Support@ons.gov.uk or visit the SRS website.

## Abbreviations

ISR: Iterative Sequential Regression
GAM: Generalised Additive Model
SARS-CoV-2: Severe acute respiratory syndrome coronavirus 2.
