# Detecting changes in population trends in infection surveillance using community SARS-CoV-2 prevalence as an exemplar

**DOI:** 10.1093/aje/kwae091

**Published:** 2024-05-29

**Authors:** Emma Pritchard, Karina-Doris Vihta, David W Eyre, Susan Hopkins, Tim E A Peto, Philippa C Matthews, Nicole Stoesser, Ruth Studley, Emma Rourke, Ian Diamond, Koen B Pouwels, Ann Sarah Walker, COVID-19 Infection Survey Team

**Affiliations:** Nuffield Department of Medicine, University of Oxford, Oxford OX3 7BN, United Kingdom; NIHR Health Protection Research Unit in Healthcare-Associated Infections and Antimicrobial Resistance, Nuffield Department of Medicine, University of Oxford, Oxford OX3 9DU, United Kingdom; Nuffield Department of Medicine, University of Oxford, Oxford OX3 7BN, United Kingdom; NIHR Health Protection Research Unit in Healthcare-Associated Infections and Antimicrobial Resistance, Nuffield Department of Medicine, University of Oxford, Oxford OX3 9DU, United Kingdom; Department of Engineering Science, Mathematical, Physical and Life Sciences Division, University of Oxford, Oxford OX1 3PJ, United Kingdom; NIHR Health Protection Research Unit in Healthcare-Associated Infections and Antimicrobial Resistance, Nuffield Department of Medicine, University of Oxford, Oxford OX3 9DU, United Kingdom; NIHR Oxford Biomedical Research Centre, University of Oxford, Oxford OX3 9DU, United Kingdom; Big Data Institute, Nuffield Department of Population Health, University of Oxford, Oxford OX3 7LF, United Kingdom; NIHR Health Protection Research Unit in Healthcare-Associated Infections and Antimicrobial Resistance, Nuffield Department of Medicine, University of Oxford, Oxford OX3 9DU, United Kingdom; Healthcare-Associated Infection and Antimicrobial Resistance Division, UK Health Security Agency, London NW9 5EQ, United Kingdom; NIHR Health Protection Research Unit in Healthcare-Associated Infections and Antimicrobial Resistance, Imperial College London, London SW7 2AZ, United Kingdom; Nuffield Department of Medicine, University of Oxford, Oxford OX3 7BN, United Kingdom; NIHR Health Protection Research Unit in Healthcare-Associated Infections and Antimicrobial Resistance, Nuffield Department of Medicine, University of Oxford, Oxford OX3 9DU, United Kingdom; NIHR Oxford Biomedical Research Centre, University of Oxford, Oxford OX3 9DU, United Kingdom; Department of Infectious Diseases and Microbiology, Oxford University Hospitals NHS Foundation Trust John Radcliffe Hospital, Oxford OX3 9DU, United Kingdom; Nuffield Department of Medicine, University of Oxford, Oxford OX3 7BN, United Kingdom; Francis Crick Institute, London NW1 1AT, United Kingdom; Division of Infection and Immunity, University College London, London WC1E 6BT, United Kingdom; Department of Infection, University College Hospital, University College London Hospitals NHS Foundation Trust, London NW1 2BU, United Kingdom; Nuffield Department of Medicine, University of Oxford, Oxford OX3 7BN, United Kingdom; NIHR Health Protection Research Unit in Healthcare-Associated Infections and Antimicrobial Resistance, Nuffield Department of Medicine, University of Oxford, Oxford OX3 9DU, United Kingdom; NIHR Oxford Biomedical Research Centre, University of Oxford, Oxford OX3 9DU, United Kingdom; Department of Infectious Diseases and Microbiology, Oxford University Hospitals NHS Foundation Trust John Radcliffe Hospital, Oxford OX3 9DU, United Kingdom; Office for National Statistics, Newport NP10 8XG, United Kingdom; Office for National Statistics, Newport NP10 8XG, United Kingdom; Office for National Statistics, Newport NP10 8XG, United Kingdom; NIHR Health Protection Research Unit in Healthcare-Associated Infections and Antimicrobial Resistance, Nuffield Department of Medicine, University of Oxford, Oxford OX3 9DU, United Kingdom; Health Economics Research Centre, Nuffield Department of Population Health, University of Oxford, Oxford OX3 7LF, United Kingdom; Nuffield Department of Medicine, University of Oxford, Oxford OX3 7BN, United Kingdom; NIHR Health Protection Research Unit in Healthcare-Associated Infections and Antimicrobial Resistance, Nuffield Department of Medicine, University of Oxford, Oxford OX3 9DU, United Kingdom; NIHR Oxford Biomedical Research Centre, University of Oxford, Oxford OX3 9DU, United Kingdom

**Keywords:** change-point detection, SARS-CoV-2 infection, community surveillance, real-time monitoring

## Abstract

Detecting and quantifying changes in the growth rates of infectious diseases is vital to informing public health strategy and can inform policymakers’ rationale for implementing or continuing interventions aimed at reducing their impact. Substantial changes in SARS-CoV-2 prevalence with the emergence of variants have provided an opportunity to investigate different methods for doing this. We collected polymerase chain reaction (PCR) results from all participants in the United Kingdom’s COVID-19 Infection Survey between August 1, 2020, and June 30, 2022. Change points for growth rates were identified using iterative sequential regression (ISR) and second derivatives of generalized additive models (GAMs). Consistency between methods and timeliness of detection were compared. Of 8 799 079 study visits, 147 278 (1.7%) were PCR-positive. Change points associated with the emergence of major variants were estimated to occur a median of 4 days earlier (IQR, 0-8) when using GAMs versus ISR. When estimating recent change points using successive data periods, 4 change points (4/96) identified by GAMs were not found when adding later data or by ISR. Change points were detected 3-5 weeks after they occurred under both methods but could be detected earlier within specific subgroups. Change points in growth rates of SARS-CoV-2 can be detected in near real time using ISR and second derivatives of GAMs. To increase certainty about changes in epidemic trajectories, both methods could be used in parallel.

## Introduction

Infectious disease surveillance has 2 broad goals: (1) identifying outbreaks which lead to sudden changes in incidence/prevalence and (2) detecting the emergence of more virulent/resistant strains of the pathogen. Reasons for changes in infectious disease trends vary—for example, changing population susceptibility causing increasing group A streptococcal infections^1^; emerging antimicrobial-resistant strains, such as ribotype-027 *Clostridium difficile* or Gram-negative pathogens carrying extended-spectrum β-lactamases^2^; and mutations affecting transmissibility in COVID-19.^3^ While laboratory sequencing methods can accurately identify variants,^4^ limited sampling and resources mean that retrospective statistical models are often more practical for monitoring infectious diseases, particularly with increasing availability of linked electronic health records.

While change-point detection methods are numerous, many are suboptimal for use on infectious disease time series in near real time. Statistical methods involve locating points in a time series where some property of the data (eg, distribution, scale) changes.^5^ Given the epidemiologic drivers above, identifying points at which the rate of change in the trend is increasing/decreasing is most useful in infectious disease surveillance. However, many statistical methods identify step changes, that is, changes in mean levels in a time series, rather than the more gradual trend changes^6^ characteristic of changing infectious disease epidemiology. Other methods require prespecifying the number of change points, and many are computationally expensive and therefore not practical for near real-time use. During the COVID-19 pandemic, while studies used change-point analysis to retrospectively assess the impact of interventions (eg, lockdowns and gatherings),^7^^‑^^9^ change-point detection methods for near real-time use have been less commonly assessed.^10^

Two methods that consider more gradual changes and find change points in trends are iterative sequential regression^11^^,^^12^ (ISR) and second derivatives of generalized additive models (GAMs).^13^ While both have been evaluated separately,^11^^,^^14^ to our knowledge they have never been directly compared. ISR provides a clear statistical assessment of when rates change and estimates constant growth rates between change points, potentially maximizing power when this is close to true underlying trends. However, it considers data sequentially, fixing change points as it iterates, thus not necessarily optimizing overall fit. Second derivatives of GAMs have been used to identify periods of change^14^^,^^15^ and to quantify change points.^13^ Their flexibility allows estimates to closely reflect reality, but the extent to which smoothing through penalized splines reduces the ability to detect change points in near real time is unclear.

We aimed to compare the performance of GAMs and ISR for change-point detection for infectious disease surveillance, both retrospectively and in near real time, using COVID-19 as an exemplar for surveillance more generally—for example, using linked electronic health records. Rapid changes in SARS-CoV-2 prevalence coupled with multiple emerging variants over the course of the COVID-19 pandemic have provided an ideal opportunity to test these methods in real-world data which are more complex than simulations. We compared the consistency and timeliness of detection between ISR and second derivatives of GAMs for identifying changes in growth rates of SARS-CoV-2 positivity over time using the United Kingdom’s Office for National Statistics (ONS) COVID-19 Infection Survey. We assessed whether earlier detection was possible considering positivity separately by age group or by available proxies for viral variant.

## Methods

### Study design

The ONS COVID-19 Infection Survey was a large household survey with longitudinal follow-up. Private households were continuously selected randomly from address lists (nonresponse in^16^) and previous ONS surveys to provide a broadly representative sample across the United Kingdom^17^ (see Supplementary Tables 3-6 in Vihta et al^18^). Following verbal consent, study workers visited each household to take written informed consent for individuals aged ≥2 years (from parents/caregivers for children aged 2-15 years; those aged 10-15 years also provided written assent). The study received ethical approval from the South Central Berkshire B Research Ethics Committee. At the first study visit, participants were asked for consent to receive optional follow-up visits weekly for the next month, and then monthly thereafter (>98.5% provided such consent). At each visit, participants provided a nose and throat self-swab and completed questionnaires.^19^

### Study population

Analysis included all visits with positive or negative swabs from August 1, 2020, to June 30, 2022 (*n* = 225 348 [2%] visits, with void/missing results excluded).

### Statistical analyses

Our outcome measure was the proportion of study visits with polymerase chain reaction (PCR)-positive SARS-CoV-2 tests. We compared 2 methods for detecting changes in trend over time: ISR^11^ and second derivatives of GAMs.^13^ All analyses were conducted separately for 12 geographical regions (9 English regions and 3 devolved administrations: Wales, Scotland, and Northern Ireland) due to positivity trend differences. ISR estimates change points in a single time series; separate GAMs by region differed only slightly from GAMs including region × time interactions, and reduced computational time (Figure S1).

ISR, using a negative-binomial distribution with a log link allowing overdispersion, initially fitted a log-linear trend within the first month’s data to September 1, 2020. Three days’ data were sequentially added to the time series, fixing change points if a new trend reduced the Akaike information criterion (AIC) by ≥6.635 points (critical value at *P* =.01, to reduce the impact of false-positives). If a change point was fixed, a new change point was not considered in the subsequent 7 days. Change points and dates’ change points were permanently fixed into the model (“detection date”) were extracted from fitted models (Appendix S1).

GAMs, using a negative-binomial distribution with a log link, included a single explanatory variable of time (in days) since August 1, 2020, and were modeled using thin plate splines.^20^ The number of basis functions, *k*, determines the flexibility of the model. The number of basis functions was selected from the series 25, 50, 75, 100, choosing the lowest value with predicted positivity within ±0.25% (absolute scale) versus *k* = 100, optimizing computational time, without large increases in the effective number of degrees of freedom^21^ (Figure S2). Splines were penalized on the basis of the third derivative, as the second derivative was the measure of interest.

Derivatives were estimated for smooth functions using posterior simulation with a Metropolis-Hastings sampler (as implemented in the gam.mh function from the *mgcv* R package),^22^^,^^23^ since standard Gaussian approximation will be poor in low-positivity periods (details are given in Appendix S1). Software code was adapted from the *derivatives* function in the R *gratia* package, which currently can only obtain derivatives on the linear predictor scale.^24^ Change points were defined at the first day on which zero was excluded from the 95% credible interval of the second derivative, corresponding to 97.5% probability of change, from September 1, 2020, onwards. Positivity trends over the full time series were compared between ISR and GAMs. Change points were classified as found by both methods if they were within ±7 days of each other, an arbitrary but pragmatic window based on the distribution of time between change points identified by both methods and the timeliness of public health responses (Appendix S1, Figure S3). Change points corresponding to the emergence of the Alpha, Delta, Omicron BA.1, and Omicron BA.2 SARS-CoV-2 variants were compared between methods.

Because ISR fixes change points once found and adds data progressively, it does not need to be run on segments of data sequentially to assess near real-time detection. In near real time, one could run ISR from the latest detected change point onwards to decrease fitting time, albeit change points may differ slightly from models incorporating the full time series, as previous data can affect the AIC. To compare near real-time detection between methods, we conducted GAM analyses successively adopting a sliding-window approach. Sliding-window length was determined by running GAM analyses on shorter periods (16, 24, and 32 weeks) and assessing whether similar change points were found in the final 8 weeks, since most recent changes are of most interest in near real time. Starting from October 1, 2020 (including data from August 1, 2020), 7-day increments of data were added until the sliding-window length was reached, from which 7 days of data were removed from the start of the time series each time 7 days were added on. We selected *k* as before for sliding-window length, scaling *k* down proportionally for the shorter time series. We assessed whether all change points identified in the last 8 weeks of each model were detected within ±7 days in 5 subsequent models and/or by ISR. Due to long run times (approximately 12-36 hours per region, including estimation of derivatives), we compared GAM detection dates for the largest (London) and smallest (Northern Ireland) regions. For each change point identified in the GAM including data from the full time series, we defined the “detection date” as the last date included in the earliest successive GAM which also confirmed the change point within ±7 days. We compared change points identified in the last 4 weeks of the weekly successive GAMs with change points found in the full time-series GAM to quantify the false-positivity and -negativity rates for successive GAMs to identify recent change points.

Using the second derivative of GAMs risks potentially missing change points if positivity decreases and increases at the same rate over a short time period. While the second derivative will be significantly different from zero, new change points will not be found when positivity changes direction, as the second derivative may not cross zero. We summarized the number and position of additional change points added if placed where, over periods of second-derivative significance, the first derivative changed from significantly positive to negative or vice versa.

### Sensitivity analysis

To assess whether earlier detection of change points was possible by focusing on high-risk population subgroups, change points estimated from separate ISR and GAMs in 3 age groups (age 2 years to school year 11 [approximately age 16 years], school year 12 to age 49 years, and age ≥50 years) were compared with combined all-age estimates. We considered separate analysis by PCR gene positivity as a proxy for SARS-CoV-2 variant—Delta and BA.2 being spike protein (S) gene target–positive (SGTP), whereas Alpha and BA.1 had S-gene target failure (SGTF). Model analyses were carried out separately with SGTP and SGTF positivity designated as outcomes, with all other positives (including those positive on only the nucleoprotein (N) gene or open reading frames 1a and 1b [ORF1ab]) being in the negative comparator group, comparing change points with the “all positives” model.

All analysis was conducted in R, version 4.0.2.

## Results

**Figure 1:**
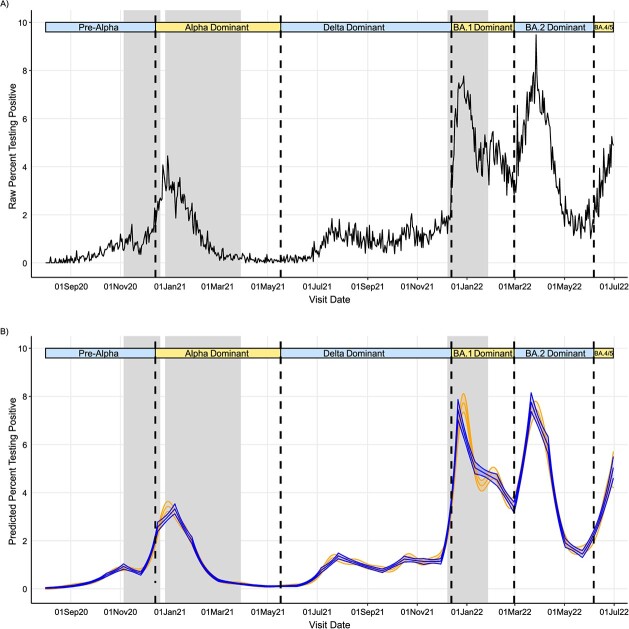
Raw percentage of nasal and throat swabs testing SARS-CoV-2–positive (A) and predicted percentage of study visits testing SARS-CoV-2–positive (B) using iterative sequential regression (blue curves) and second derivatives of generalized additive models (orange curves) in the London region, COVID-19 Infection Survey, August 2020-June 2022. The vertical dashed lines indicate periods when new variants became dominant, defined as more than 50% of positive swabs with a cycle threshold less than 30 being S-gene target–positive (ORF1ab + N + S, ORF1ab + S, N + S gene positivity) in the COVID-19 Infection Survey for the pre–Alpha variant period (August 1, 2020-December 13, 2020), the Delta variant period (May 17, 2021-December 12, 2021), and the Omicron BA.2 variant period (February 28, 2022-June 5, 2022) and more than 50% cycle threshold less than 30 S-gene target–negative (ORF1ab + N gene positivity) for the Alpha variant period (December 14, 2020–May 16, 2021), the Omicron BA.1 variant period (December 13, 2021-February 27, 2022), and the Omicron BA4/BA.5 variant period (June 6, 2022, onwards). Gray shading indicates periods in which stay-at-home/work-from-home laws were enforced, although specific restrictions varied across the time series. N, nucleoprotein; ORF1ab, open reading frames 1a and 1b; S, spike glycoprotein.

From August 1, 2020, to June 30, 2022, a total of 8 799 079 study visits from 533 157 participants in 266 400 households returned 147 278 (1.7%) SARS-CoV-2–positive swabs (characteristics are shown in Table S1). From August to November 2020 (pre-Alpha variant), positivity rose to approximately 1%, before increasing to approximately 2% in January 2021 (Alpha variant; Figure 1A, Figure S4A). Positivity decreased until June 2021 before increasing to approximately 1%-2% in July-December 2021 (Delta variant). Positivity rose sharply to approximately 6% from December 2021 onward (BA.1), decreasing to approximately 3.5% by February 2022, before increasing to approximately 7.5% by mid-March (BA.2). Rises with the BA.4/BA.5 variant began in June 2022. During the pre-Alpha period, 10% of strong positives (cycle threshold < 30) had SGTF versus 79%, 1%, 84%, and 9% in Alpha-, Delta-, BA.1-, and BA.2-dominant periods (Table S2). Positivity varied by region, particularly between northern/southern English regions—for example, higher positivity pre-Alpha in Yorkshire than in London (Figure S4B).

### Detecting changes in growth rates using ISR and GAMs

**Figure 2:**
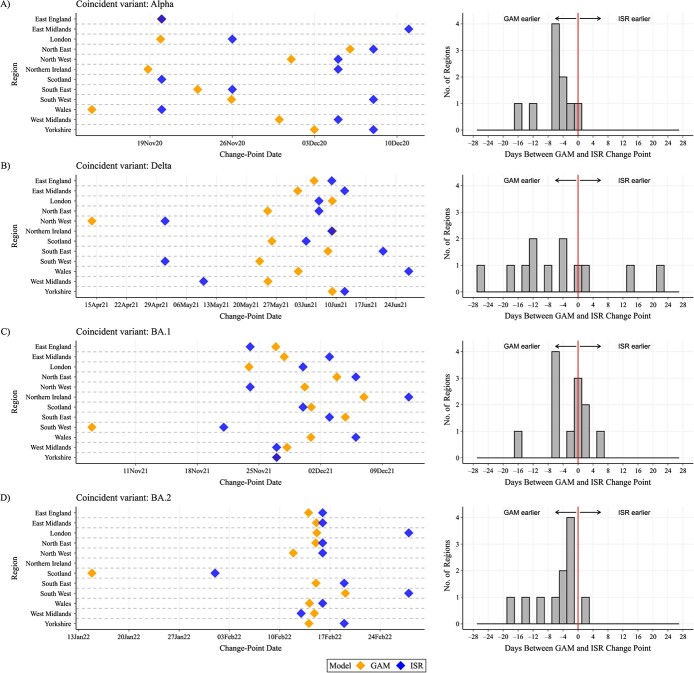
Change points in SARS-CoV-2 prevalence corresponding to the emergence of 4 key SARS-CoV-2 variants found by iterative sequential regression (ISR) and second derivatives of generalized additive models (GAMs), COVID-19 Infection Survey, August 2020-June 2022. Results are shown for the Alpha variant (A), the Delta variant (B), the Omicron BA.1 variant (C), and the Omicron BA.2 variant (D). ISR and GAM analyses were carried out separately for each of the 12 geographical regions presented, and all were conducted on the full time series between August 2020 and June 2022. In panel A, for East Midlands and Scotland there was no change point coincident with the Alpha variant using GAMs. In panel D, for Northern Ireland there was no change point coincident with the BA.2 variant for GAMs and ISR. Exact dates of the change points are shown in Table S3.

We compared change points detected via the 2 methods, first considering the emergence of dominant SARS-CoV-2 variants. ISR and GAMs made similar predictions of changing positivity trends across geographical regions over the study period (Figure 1B, Figure S5). In London, change points corresponding to the emergence of the Alpha, Delta, BA.1, and BA.2 variants occurred on November 26, 2020, June 6, 2021, November 30, 2021, and February 28, 2022, using ISR (Figure 2, Table S3) and 6 days earlier, 3 days later, 6 days earlier, and 13 days earlier, respectively, using GAM derivatives. Across all regions, change points for the 4 variants occurred a median of 4 days earlier (IQR, 0-8; range, 22 days later to 26 days earlier) with GAMs versus ISR. Sixty-nine percent (33/48) of change points occurred earlier using GAMs. No change point was detected for the Alpha variant in the East Midlands or Scotland using GAMs, but change points were detected using ISR.

Both methods identified other change points aside from trend increases resulting from emerging variants, described for London in Table 1. Sixty-three percent (12/19) of all change points in London identified in GAMs were identified using ISR within ±7 days. Fifty-seven percent (12/21) of all change points in London identified by ISR were identified by GAMs within ±7 days. Inconsistent change points between methods generally reflected small fluctuations when positivity was low. Thirty-three percent of change points representing increasing positivity from GAMs and ISR were followed by relative percentage positivity increases greater than 150% (Appendix S2, Figure S6).

**Table 1:** All change points in SARS-CoV-2 prevalence found by iterative sequential regression and second derivatives of generalized additive models for the London region of the COVID-19 Infection Survey, United Kingdom, September 2020–June 2022^a^

**GAM change-point date**	**ISR change-point date**	**Description of trend**	**Time difference between ISR and GAM change points, d** ^**b**^
September 26, 2020		Faster growth	
	October 15, 2020	Faster growth	
November 2, 2020	November 5, 2020	Increase to decrease	−3
November 20, 2020	November 26, 2020	Decrease to increase (rise in Alpha variant)	−6
December 19, 2020	December 17, 2020	Slower growth (slowing down of Alpha variant)	2
	January 7, 2021	Increase to decrease	
January 23, 2021	January 28, 2021	Faster decline	−5
February 5, 2021		Slower decline	
	March 2, 2021	Slower decline	
	May 1, 2021	Decrease to increase	
June 9, 2021	June 6, 2021	Faster growth (rise of Delta variant)	3
July 12, 2021	July 6, 2021	Slower growth	6
	July 27, 2021	Increase to decrease	
September 25, 2021	September 19, 2021	Decrease to increase	6
October 15, 2021	October 16, 2021	Increase to decrease	−1
November 1, 2021		Decrease to increase	
	November 9, 2021	Decrease to increase	
November 24, 2021	November 30, 2021	Faster growth (rise of BA.1 variant)	−6
December 20, 2021	December 21, 2021	Increase to decrease (decline of BA.1 variant)	−1
January 6, 2022	January 11, 2022	Slower decline	−5
January 29, 2022		Slower decline	
	February 7, 2022	Decrease to increase	
February 15, 2022		Faster growth (rise of BA.2 variant)	
	February 28, 2022	Decrease to increase (rise of BA.2 variant)	
March 16, 2022	March 21, 2022	Increase to decrease (decline of BA.2 variant)	−5
	April 11, 2022	Faster decline	
April 19, 2022		Faster decline	
	May 2, 2022	Slower decline	
May 26, 2022	May 23, 2022	Decrease to increase (rise of BA.4/BA.5 variant)	3

Abbreviations: ISR, iterative sequential regression; GAM, generalized additive model.

^a^ Change points were classified as found by both models if they were within ±7 days of each other. If a corresponding change point was not identified by either GAMs or ISR, cells in the Table were left blank and a time difference between change points could not be calculated.

^b^ Negative numbers indicate earlier occurrence of change points using GAMs, as compared with ISR.

### Detecting change points in “near real time”

While retrospectively detecting change points can quantify how epidemic growth has varied, ideally change points would be detected in near real time to inform measures intended to control growth. Comparing GAM analyses conducted on double (16 weeks), triple (24 weeks), or quadruple (32 weeks) an arbitrary but realistic 8-week period of interest showed that 32 weeks’ data was the minimum amount that avoided missing over half the change points in the full time series (Table S4, Appendix S2).

**Figure 3:**
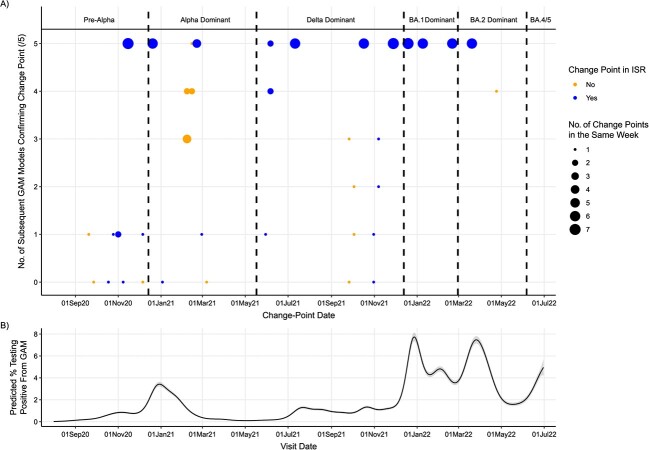
Change points in SARS-CoV-2 prevalence found by generalized additive model (GAM) analyses run successively over 32-week periods from September 2020 to June 2022 for the London region of the COVID-19 Infection Survey, according to the number of successive GAMs each change point was confirmed by (range, 0-5), and whether the change points were identified by iterative sequential regression (ISR) (A). Change points in the same week (starting Monday) found in the same number of subsequent models were grouped together (indicated by size of circle). Points are blue if at least 1 change point in that week was also found by ISR, and points are orange if no change points in that week were found by ISR. The lower graph (B) shows the predicted positivity from the final GAM for reference. Gray shading indicates the 95% credible interval.

When we conducted GAM analyses successively adding new data for London every week from October 1, 2020, through June 30, 2022, we found 96 change points in the final 8 weeks across all GAMs (Figure 3). Most (64/96 [67%]) change points were identified by 5 successive GAMs. Eight (8%) change points were not identified in any of the 5 subsequent GAMs, but 4 of these were identified by ISR. Overall, 77% (74/96) of the change points in the last 8 weeks of successive GAMs were identified by ISR, and 23% (22/96) were never identified by ISR. Results were similar for Northern Ireland (Appendix S2, Figure S7). Change points in the final 4 weeks of successive GAMs found 62% (8/13) of change points in the full time-series GAM, including for all major variant increases (Table S5, Appendix S2).

Using the final date of the first successive GAM to estimate when change points in the full time-series GAM would have been detected in near real time, for London, change points were detected a median of 21 (IQR, 17-26; range, 10-128) days later (Figure 4, Table S6). ISR generally fixed change points into the model (based on lower AIC vs linear trend) 24 days after the change. When identified by both GAMs and ISR, successive GAMs detected change points a median of 4 (IQR, 10 days earlier to 1 day later; range, 17 days earlier to 35 days later) days earlier. Four change points identified in the final GAM for London were not identified in any successive GAMs; hence detection dates could not be determined.

**Figure 4:**
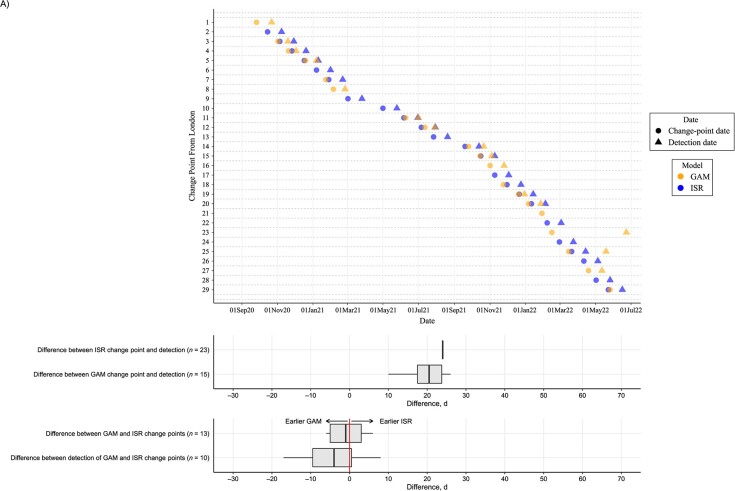
Continues

**Figure 4:**
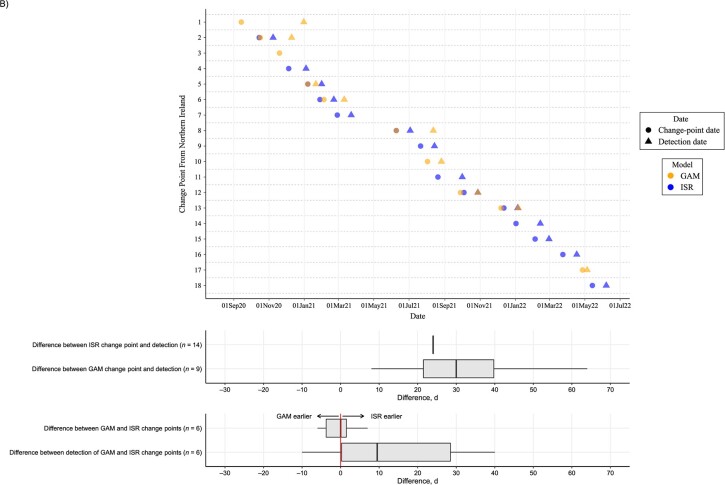
Dates of change points in SARS-CoV-2 prevalence (circles) and the date on which the change point would have first been detected in near-real time (detection dates; triangles) from generalized additive models (GAMs; orange) and iterative sequential regression (ISR; blue) for the London (A) and Northern Ireland (B) regions of the COVID-19 Infection Survey, September 2020-June 2022. Box plots show the median (vertical line inside the box) and IQR (indicated by the 25th and 75th percentiles at the edge of the box) number of days between ISR and GAM change points and their detection dates.The whiskers of the box plot extend to 1.5 times the IQR from the quartiles, unless the data's minimum or maximum values fall within this range, in which case the whiskers are set at the actual minimum or maximum values.

When considering change points for Northern Ireland (approximately one-fifth of the visits from London), while ISR still detected change points approximately 24 days after the change occurred, GAMs detected changes a median of 30 (IQR, 24-54; range, 8-108) days later (Figure 4, Table S6). When identified by both ISR and GAMs, in contrast to London, ISR detected change points a median of 10 (IQR, 0-32) days earlier.

### Incorporating change points based on the first derivative

Change points for BA.4/BA.5 were found for all regions using ISR, but were not found using GAMs for 9 of the 12 regions (Table S7). The BA.4/BA.5 growth rate was similar to BA.2 decline, so, while the second derivative was significantly different from zero, new change points were not established. Adding in additional change points where the first derivative switched signs, GAMs found change points for BA.4/BA.5 in all regions (Figure S8). See Appendix S2 for further details on additional change points established by first derivatives.

### Estimating change points in target subgroups

Analogous to “sentinel surveillance,” we assessed whether change points could be established earlier by modeling population subgroups—here, age. In our dataset, as in others,^17^ large rises in positivity associated with Alpha variant emergence occurred earlier in the age 2 years–school year 11 group, with steeper increases in positivity in late August 2021 (Delta) and late January 2022 (BA.1) versus older age groups (Figures S9 andS10).

**Figure 5:**
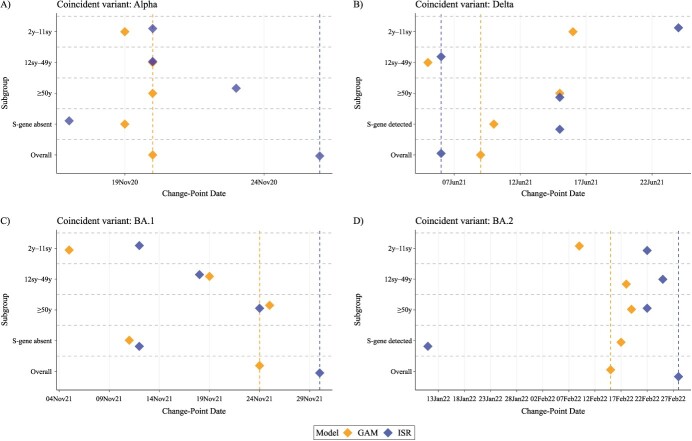
Change points in SARS-CoV-2 prevalence from generalized additive model (GAM; orange) and iterative sequential regression (ISR; blue) analyses conducted separately by age group (defined by years (y) and/or school year (sy)), spike glycoprotein (S) gene detection and overall, in the London region of the COVID-19 Infection Survey. Results are shown for the Alpha variant (A), the Delta variant (B), the Omicron BA.1 variant (C), and the Omicron BA.2 variant (D). All models included data from the full time series (August 2020-June 2022). Vertical dashed lines show the position of change points in overall GAM and ISR analyses. Change-point dates are provided in Table S8.

Little difference was seen across age groups for GAM change points associated with the Alpha variant (Figure 5). For the Delta variant, change points occurred earliest in the overall model and the school year 12–age 49 years group and latest in the age 2 years–school year 11 group. Rises in BA.1 occurred 18 days earlier in the youngest age group versus all ages using ISR, and 19 days earlier using GAMs. Rises in BA.2 were found earliest in the age 2 years–school year 11 group using GAMs (February 9, 2022).

### Estimating change points by outcome type

Analogous to surveillance of different infection types (eg, resistant vs susceptible *Staphylococcus aureus*), we considered whether change points could be established earlier by modeling PCR S-gene positivity as a proxy for SARS-CoV-2 variant. There were distinct trend differences between SGTF and SGTP positivity over time (Figures S11 and S12), and GAM and ISR predictions for London closely followed these (Figure S13).

For London, change points associated with Alpha emergence occurred 1 day and 9 days earlier using SGTF versus all positives for GAMs and ISR, respectively (Figure 5). Change points for Delta occurred and were detected 9 days later using SGTP versus all positives using ISR, and occurred 1 day later using GAMs. Change points for BA.1 occurred on November 11 and 12, 2021, using GAMs and ISR for SGTF, with all-positive change points on November 24 and 30, 2021, respectively, a 15-day earlier detection for ISR. For SGTP, ISR estimated a change point for BA.2 on January 11, 2022 (detected February 4, 2022) and did not find a change point for all positives until 48 days later.

## Discussion

Here, we compared 2 methods for detecting changes in growth rates in surveillance data, using SARS-CoV-2 as an exemplar. Both methods detected trend increases and decreases associated with the Alpha, Delta, Omicron BA.1, and Omicron BA.2 variants, and other smaller growth rate fluctuations, at similar dates. Considering near real-time analysis, most recent change points detected using GAMs were found in successive GAMs including 5 subsequent weeks’ data and using ISR, demonstrating consistency between GAM model runs and between methods. However, GAMs needed at least 4 times the duration of data over which there was interest in identifying change points to provide stable estimates. Change points were, on average, detected slightly earlier using GAMs versus ISR considering larger geographical regions, but this was not consistent across different-sized regions or subgroups. Considering positivity trends separately in different age subgroups allowed earlier detection of the BA.1 variant using data from children alone versus all positives and separately for SGTP of the BA.2 variant.

Supporting the use of these methods for sentinel surveillance, we found that change points could be detected earlier through modeling of different age groups separately—often, but not always, in the age 2 years–school year 11 group. This is likely due to faster SARS-CoV-2 transmission at younger ages, specifically during BA.1 variant emergence, driven by higher contact levels through school attendance while other guidance (eg, working from home^25^) slowed transmission between adults until infections were later transmitted onwards from younger persons to older individuals. While studies have found no evidence of increased transmission on school premises,^26^^‑^^29^ increased person-to-person contact associated with attending school (eg, use of public transportation, gatherings at school at pick-up/drop-off times) measurably affects the reproduction number.^30^ Rising SARS-CoV-2 positivity in younger individuals may therefore be a useful early warning signal for rises in older individuals, among whom hospitalization risk and mortality are higher,^31^ although trend changes were not consistently identified earlier in younger age groups. Implementing surveillance systems separately by subgroup may be an efficient way to detect changes earlier more generally.

We found that change points were generally estimated to occur slightly earlier when considering positivity split by S-gene detection. This was particularly useful when the BA.2 variant emerged, as BA.1 declines concealed fast BA.2 growth when combining all positives. More broadly, surveillance of pathogens with different susceptibilities could allow similar shifts in underlying variants to be elucidated.^32^

The methods have much wider applicability to infection surveillance, but SARS-CoV-2 provided an ideal opportunity to test them due to rapid changes in positivity and emerging variants with different epidemiology. Change points estimated for Alpha using ISR and GAMs were generally consistent with changing UK public health policy. The first Alpha sequence came from a sample taken on September 20, 2020, but it was not widely recognized until December after its rapid growth throughout November,^33^^,^^34^ with regional lockdowns implemented on December 23, 2020.^35^ By then, change points had been detected via ISR in 4 geographical regions, including London and South East England, where Alpha rose earliest and fastest. In contrast, Delta was named a variant of concern on May 6, 2021,^36^ approximately a month earlier than change points that occurred in most regions in our analysis, reflecting its earlier identification through rapid increases in numbers of infections in India. The appropriate length of time between changes’ occurring and change-point detection thus depends on the surrounding context. The average 3-week lag observed here may not be generalizable under large-scale specific testing, but it may be relevant if surveillance is reliant on passive data. The infection/disease being monitored could also influence the relevance of this lag. Fundamentally ISR and GAMs identify when infection epidemiology changes, which may be independent of or coincident with recognition of new variants with different transmission potential, virulence, or resistance through genetic sequencing or changes in epidemiology in other countries. While our methods could be applied to the proportion of genetic sequences which are a specific variant, to date this has generally shown log-linear growth for SARS-CoV-2,^37^ without change points before a new variant becomes the majority sequence.

Real-time surveillance is mostly concerned with recent data, where uncertainty is greatest. Using ISR, most change points were detected slightly later—a limitation of the fact that ISR requires a minimum number of days between the current and last identified change points. While GAMs detected some change points earlier, they also found a small number of change points during the last 7 days of successive model runs which were not confirmed when adding a further 7 days’ data. The increased flexibility afforded through GAMs may therefore cause false-positives at data boundaries. Most change points found in the last 4 weeks of successive GAMs were confirmed by the full time-series GAMs, albeit sometimes later due to reduced power over shorter time frames. Some change points near the end of successive model runs were not found in the full time-series GAM, possibly due to the full time-series GAMs’ oversmoothing across periods of variation. Requiring at least 7 days of data after change points or confirmation in 2 successive models would increase certainty. Change points were found slightly later with GAMs than with ISR in smaller datasets, likely due to ISR’s fixing one parameter at a time whereas GAMs optimize over the entire time series, hence being more influenced by sample size. Overall, this illustrates the inherent trade-offs between the two methods; ISR’s forcing of log-linear trends between change points will identify change points more efficiently when this is close to the truth, but will be inefficient if trends are volatile.

While most change points found in this study were related to increases and decreases in major variants, we also identified other fluctuations. Most of these other change points were associated with large relative percentage changes in positivity over 4 weeks, with most smaller relative changes indicating growth/decline slowing down or flattening off (so still being epidemiologically important). Some change points identified by GAMs were significant for short durations (eg, 1 day) and of small magnitude in the second derivative. While statistically significant, these changes may not be meaningful, with policy decisions more likely made on larger changes in growth/decay. For both GAMs and ISR, we would recommend interpreting change points in the context of current underlying prevalence. Further, using second derivatives of GAMs, change points for BA.4/BA.5 were mostly not found by June 30, 2022, but could be established when considering additional change points based on the first derivative swapping sign. Considering changes in the first derivative may be important to avoid missing change points moving forward. With regard to methods, estimating derivatives using a Metropolis-Hastings or similar sampler is recommended during low-prevalence periods.

In our exemplar, demonstrating that relevant change points can be detected in a randomly sampled community population is useful for future SARS-CoV-2 surveillance, as this could trigger targeted testing in different regions and/or age groups to help control spread and identify new variants,^38^^,^^39^ ultimately aiming to reduce numbers of cases/hospitalizations. The large sample size allowed sufficient power to detect change points, despite relatively low positivity rates, enabling us to compare the two methods. While SARS-CoV-2 is a respiratory virus, the methods apply more broadly to different infection surveillance data streams.

Limitations of this study include comparison of 2 methods in a single dataset, albeit including multiple change points of different magnitudes. While these methods have been evaluated independently in other datasets,^11^ further comparisons in other settings may be useful. Comparing methods in complex real-world data is practically useful, but future simulation studies could systematically evaluate statistical properties of these methods against a known “gold standard,” albeit likely based on simpler underlying trends. We matched change points within ±7 days between methods, which may have led to a small amount of misclassification. The amount of data required to detect change points will depend on the specific outcome and the speed of underlying changes, which will differ between respiratory pathogens (eg, SARS-CoV-2) and antimicrobial resistance determinants, for example. While ISR and second derivatives of GAMs are 2 options, other change-point detection methods may also be suitable.

In summary, ISR and second derivatives of GAMs could potentially detect changes in trend in multiple different types of infections in near real-time surveillance, including SARS-CoV-2, but more widely including hospital-acquired infections and antimicrobial-resistant pathogens. While both methods gave a generally consistent pattern, some known changes in the epidemiology of SARS-CoV-2 caused by different variants emerging were identified earlier by GAMs than by ISR and vice versa. Therefore, using both methods in parallel would be ideal.

## Supplementary Material

Web_Material_kwae091

## Data Availability

Deidentified study data are available for access by accredited researchers in the Office for National Statistics Secure Research Service (SRS) for accredited research purposes under part 5, chapter 5 of the United Kingdom’s Digital Economy Act 2017. For further information about accreditation, contact research.Support@ons.gov.uk or visit the SRS website. Key analysis code is available on GitHub at https://github.com/EmmaPritchard.

## Disclaimer

The views expressed in this article are those of the authors and not necessarily those of the National Health Service, the NIHR, the Department of Health, or Public Health England. The funders/sponsors did not play any role in the design and conduct of the study; the collection, management, analysis, and interpretation of the data; the preparation, review, and approval of the manuscript; or the decision to submit the manuscript for publication. All authors had full access to all data analysis outputs (reports and tables) and take responsibility for their integrity and accuracy. For the purpose of Open Access, the authors have applied a CC-BY public copyright license to any Author Accepted Manuscript version arising from this submission.
