# Supplementary material for: Detecting changes in population trends in infection surveillance using community SARS-CoV-2 prevalence as an exemplar
Source: Am J Epidemiol. 2024 May 29;193(12):1848–60. doi: 10.1093/aje/kwae091 (PMC7616874; doi:10.1093/aje/kwae091)
Supplement: Web_Material_kwae091 [file web_material_kwae091.pdf]

Supplementary Material

Detecting changes in population trends in infection surveillance using community SARS-CoV-2 prevalence as an exemplar

Emma Pritchard, Karina-Doris Vihta, David W. Eyre, Susan Hopkins, Tim E. A. Peto, Philippa C. Matthews, Nicole Stoesser, Ruth Studley, Emma Rourke, Ian Diamond, Koen B. Pouwels, Ann Sarah Walker, and the COVID-19 Infection Survey Team

Appendix S1 2
Appendix S2 3
Figures S1-S13 5
Tables S1-S8 18

Appendix S1

ISR algorithm

ISR initially fitted a linear trend on the log scale up to 1st September 2020 allowing no change-points to be found in this period. The model then considered the subsequent *n* days of the time-series, and fit two models: one extending the current trend (one-trend), the second allowing a change of trend (two-trend). If the two-trend reduced the AIC by at least 6·635, the change-point was permanently fixed in the model, otherwise the one-trend model was chosen. A minimum length of time between change-points (interval length) had to be chosen, as well as a minimum length of time between a change-point and its detection time (minimum distance). If the one-trend model was selected, the endpoint was moved forward one day and models with one extra change-point at all possible positions were fitted, with the one with the smallest AIC being identified (this could again be the model with no change-points), as well as all the models with an AIC within 6·635. The algorithm repeated this process until the end of the time-series.

Second derivative estimation by simulation

Derivatives were estimated for the smooth function using posterior simulation on the absolute scale. If positivity was relatively common throughout the entire period, coefficients from the GAM would approximately follow a multivariate normal distribution with mean vector and covariance matrix specified by the model estimates of the coefficients and their covariances, respectively.[1] Posterior simulation involves taking random draws from this distribution, whereby each draw represents a new trend that is consistent with the fitted model while also incorporating uncertainty in the estimated trend. However, this Gaussian approximation will be poor in periods where data consists of mostly zeros due to low positivity, as observed for some periods in our exemplar. To overcome this problem, we used a simple Metropolis-Hastings sampler to generate samples from the posterior distribution of the fitted model (as implemented in the gam.mh function from the *mgcv* R package).[2,3] This approach alternates fixed proposals – based on the typical Gaussian approximation to the posterior – with random walk proposals, based on a shrunken version of the approximate posterior covariance matrix. The random walk component ensures that the chain does not get stuck in regions for which the Gaussian proposal density is much lower than the posterior density.[2]

First, 2000 curves were simulated from the fitted GAMs using a Metropolis-Hastings algorithm. First and second derivatives on the absolute scale were then estimated for each simulation using backwards finite differences, allowing estimation on the final day of data (not possible with forward of central differences). The median, 2·5th, and 97·5th percentiles were estimated across the simulations, obtaining an average with credible intervals

Classifying change-points found by GAMs and ISR

For all change-points found by the GAMs for each region (n=199), we identified the closest ISR change-point and calculated the number of days between the GAM change-point and the ISR change-point. We repeated this for the ISR change-points i.e. for all change-points found by ISR for each region (n=230), we identified the closest GAM change-point, and calculated days between the ISR change-point and the GAM change-point. We plotted the number of days between change-points for each of these analyses (**Figure S3**).

For the GAM change-points, the closest ISR change-points were a median 1 day earlier (IQR 6 days earlier, 4 days later) (**Figure S3**). For ISR change-points, the closest GAM change-points were a median 1 day later (IQR 5 day earlier, 10 days later). Given this distribution, and the potential timeframe for public health responses, change-points were therefore assumed to reflect the same change in underlying trend if they were within ±7 days of each other, allowing the majority of closest change-points to be classified as reflecting the same underlying change in trend, whist also being sensitive to change-points further away in time being more unlikely to be capturing the same underlying change in the data.

Appendix S2

Relative percentage change in positivity after change-points

For each change-point identified by GAMs and ISR run on the full time-series, for each region separately, the predicted percentage testing positive on the day of each change-point was compared with the predicted percentage testing positive 4 weeks later, as estimated by each respective model. The relative percentage change was calculated between these two predicted percentages.

Across all regions, 199 change-points were identified using GAMs run on the full time-series. Of these change-points, 104 (52%) were followed by a decrease in positivity over 4 weeks, 94 (47%) were followed by an increase, and one (1%) change-point was less than 4 weeks before the end of the time series (**Figure S6**). Of those change-points followed by decreasing positivity, 62% had a relative decrease of more than 20%, and 25% had a relative decrease of more than 50%. 19% of decreasing GAM change-points had a relative decrease of between 0-10%. For change-points followed by increasing positivity, 45% had a greater than 100% relative increase in positivity over 4 weeks, with over a third having a relative increase of greater than 150%. 11% of increasing GAM change-points had a relative increase of between 0-10%.

For ISR, 230 changes-points were identified across all regions of which 108 (47%) were followed by decreasing positivity over 4 weeks and 122 (53%) were followed by an increase. Of those change-points followed by decreasing positivity, 72% decreased by more than 20% in relative terms and 31% decreased by more than 50% relatively. 14% of change-points decreased between 0-10% relatively. For change-points followed by increasing positivity, 45% had a greater than 100% relative increase 4 weeks, with over a third having a relative increase of greater than 150%. 11% of increasing GAM change-points had a relative increase of between 0-10%. For change-points followed by increasing positivity, 52% had a greater than 100% relative increase over 4 weeks, with over a third having a relative increase of greater than 150%. 8% of increasing GAM change-points had a relative increase of between 0-10%.

Detection of change-points in near real-time: running GAMs on shorter time periods

In real-time, often most interest is in change-points at the end of a time-series, for example, the final 8-weeks. Rather than running GAMs from the start of the time series (1st August 2020) each time we wanted to find new change-points at the end of the time-series, to improve computational efficiency we assessed whether the same change-points were estimated if GAMs were only run on double (16-weeks), triple (24-weeks), or quadruple (32-weeks) the period of interest. The shorter time-frames of 16-weeks and 24-weeks missed over half the change-points in the full time-series in both cases so were not considered further (**Table S4**).

In contrast, the 32-week model found the majority of change-points in the full time-series (8/10). In the 32-week model, one change-point on the 21 February 2021 was not identified by the full model in the model ending 18 March 2021. This change in the second derivative was only significant for two days, thus may not be a meaningful change. The second derivative became significant again on the 24 February 2021 for nine days, matching the change-point in the full model on the 23 February 2021. With models ending on 23 December 2021 and 17 February 2022, the 32-week model missed two change-points identified in the full time-series (4 Nov 2021, 2 Feb 2022). While these were not change-points indicating substantial growth/decay of variants, they were both identified by ISR (9 Nov 2021, 7 Feb 2022; **Table 1**). Thus, while 32-weeks appeared to identify the majority of change-points, if the capacity is available to run models on the full time-series, statistical power will likely be increased.

Detection of change-points in 'near real-time' for Northern Ireland

Comparing to Northern Ireland (the smallest region in our dataset), 52 change-points were found in the final 8-weeks across all GAMs (**Figure S7**) The majority (31/52: 60%) of these change-points were identified by five successive GAMs. One change-point was not identified in any of the five subsequent GAM models, but was identified by ISR. Overall, 52% (27/52) of change-points in the last 8-weeks of successive GAMs were identified by ISR, and 48% (25/52) were never identified by ISR.

Incorporating additional change-points based on the first derivative

Adding in additional change-points where the first derivative switched signs during a period of significance in the second derivative, an additional 78 change-points were established across the full time-series for all regions (199 change-points based on the second derivative only). The largest number of additional change-points occurred in South East England, with 10 additional change-points above the 20 original change-points established using the second derivative only (**Table S7**). The majority of the additional change-points occurred in January and February 2022, concurrent with the rise and fall of BA.1 (**Figure S8**).

Comparing change-points found in successive GAMs and change-points found in the full time-series

In order to have a fairer comparison between change-points found by the successive GAMs and change-points found in the GAMs run on the full time-series, we only considered change-points which occurred after 32-weeks of data was acquired. As we included data from 1st August 2020 onwards, only successive GAMs which included data from 13th March 2021 onwards were considered. Consequently, as we were considering change-points in the last 4-weeks of successive GAMs, change-points which occurred before 13th February 2021 (4-weeks before 13th March 2021) in the GAM run on the full time-series were not considered for this analysis.

We first considered all the change-points present in the full time-series GAM for London and summarised whether they were found in the last 4-weeks of at least one of the 32-week GAMs. Of the 13 change-points found in the GAM run on the full time-series for London, eight were found in the last 4-weeks of the relevant 32-week successive GAMs, giving a false negativity rate of 38% (5/13) for successive GAMs to identify recent change-points (**Table S5A**). There was no change-point identified from the 32-week successive GAMs that occurred within ±7 days of the change-point corresponding to the emergence of BA.1 on 24th November 2021 from the full time series, however two 32-week successive GAM models identified change-points on 2nd December 2021 (**Table S5B**). Whilst these change-points were >7 days after the change-point identified in the full GAM, they are highly likely to represent the same change in underlying trend and are likely later due to reduced power over the shorter 32-week time-series.

We next considered the change-points found in the last 4-weeks of all the 32-week GAMs and summarised whether they were found in the full time-series GAM to calculate the rate of false positives. Of the 22 change-points found in the last 4-weeks of the GAMs run successively using 32-week sliding windows, 13 corresponded to change-points found in the GAM run on the full time-series, giving a positive predictive value of 59% (13/22) (**Table S5B**). The remaining change-points identified in the successive GAMs but not the full-time series (false-positives) were mostly close to change-points identified in the full GAM but potentially not identified within 7 days due to lower power using 32-weeks of data. This included the following change-points found in the last 4-weeks of the 32-week models: 9th November 2021, 2nd December 2021, 24th February 2022, and 24 March 2022; corresponding with change-points in the full GAM on 1st November 2021, 24th November 2021, 15th February 2022, and 16th March 2022, respectively. Grouping change-points in the 32-week models with corresponding change-points in the full GAM >7-days apart (7 change-points), the rate of false positivity would instead be 9% (2/22).

Comparing change-points in the full time-series GAM to those in the 32-week successive GAMs should be interpreted cautiously for two reasons. Firstly, the GAM run on the full time-series may not be the gold standard and instead might over smooth across periods of variation. Describing change-points that are identified in the last 4-weeks of the shorter GAMs but not in the GAM run on the full time-series as "false positives" may therefore not be appropriate and could be identifying real changes smoothed out when using the longer time-series. Secondly, the 32-week models include less data than the full time-series and may therefore have lower power to detect genuine changes. When comparing to the full data, we observed some change-points in the shorter GAMs showing the same changes as were present in the full GAM, but occurring around 9 days later. With less data and thus potentially larger confidence intervals, the ±7-day time frame to classify change-points as reflecting the same underlying change in trend may not be as appropriate.

Figures S1-S13

Figure S1: Comparison of GAMs with region included at an interaction with time (red) and as separate models for each region (blue)

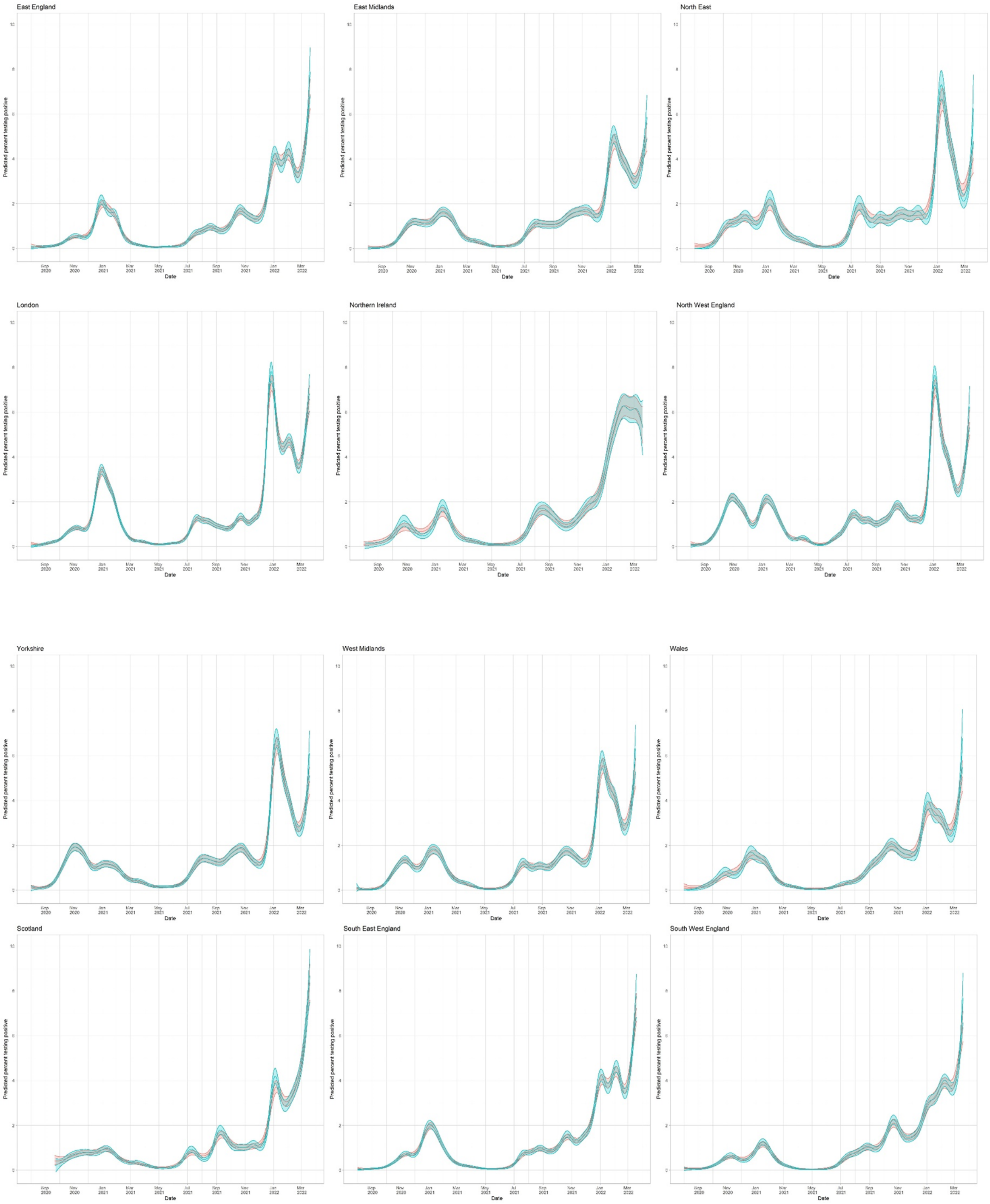

Note: The run-time for the model including the interaction between region-time was 84·6 hours, versus approximately 4 hours when running all regions separately, before derivatives were estimated.

Figure S2: Difference in predicted percentage testing positive from GAMs with varying numbers of basis functions (k) of 25, 50, 75, 100, for London only

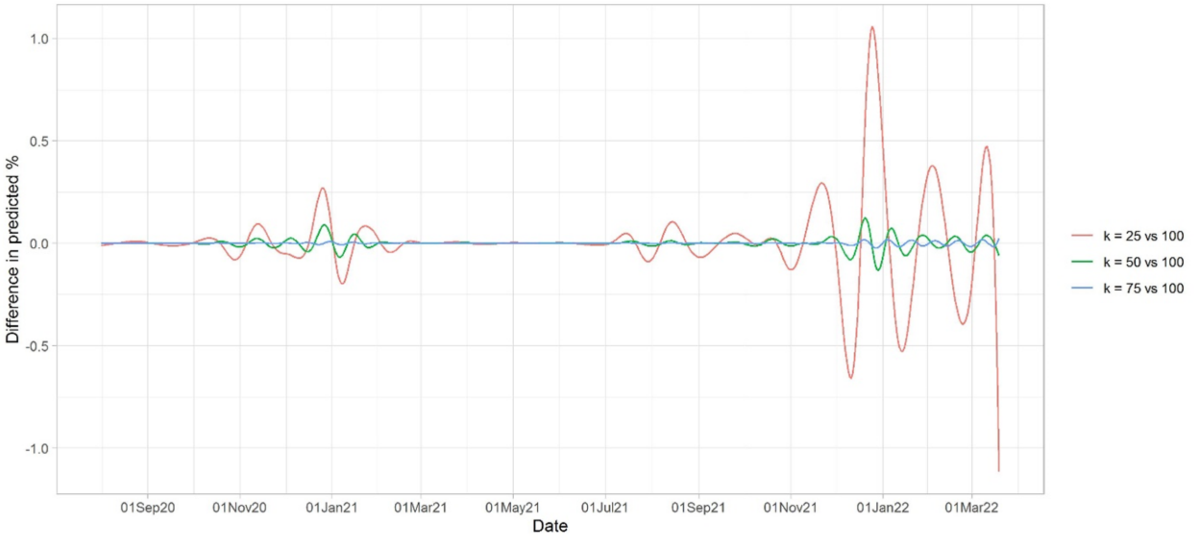

Note: The median (IQR) [range] of differences between GAMs with k = 25, 50, 75 vs k = 100 were -0·0005 (-0·08, 0·01) [-1·09, 1·79], 0·0002 (-0·007, 0·010) [-0·1287, 0·2087], and 0·000007 (-0·0015, 0·0017) [-0·036, 0·032], respectively. The effective degrees of freedom (EDF) were 23·4, 39·5, 44·6, and 45·6 for k = 25, 50, 75, and 100, respectively.

Figure S3: Distribution of the number of days between change-points identified by GAMs for all regions and the closest ISR change-point (A), and the number of days between change-points identified by ISR and the closest GAM change-point (B).

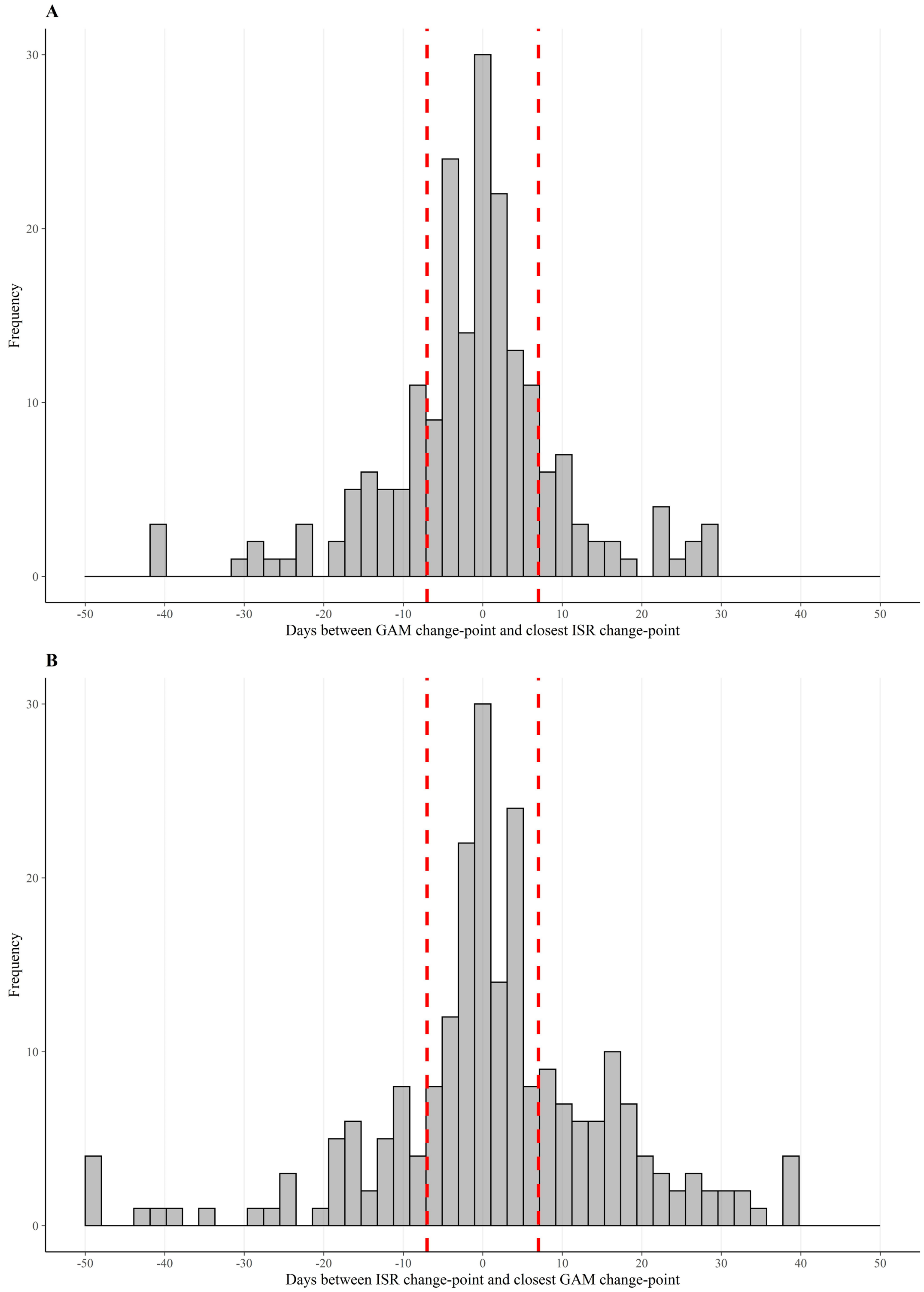

Note: Red dashed lines indicate ±7 days

Figure S4: Raw daily percentage of visits with a SARS-CoV-2 positive test over the study period overall (A) and split by region (B)

A: Raw percentage testing positive for all regions combined

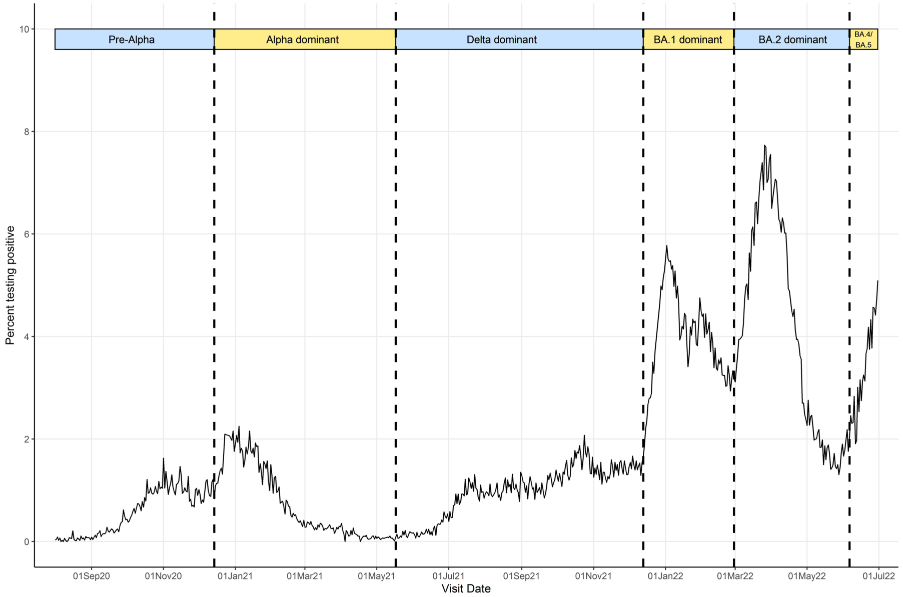

B: Raw percentage testing positive for each region

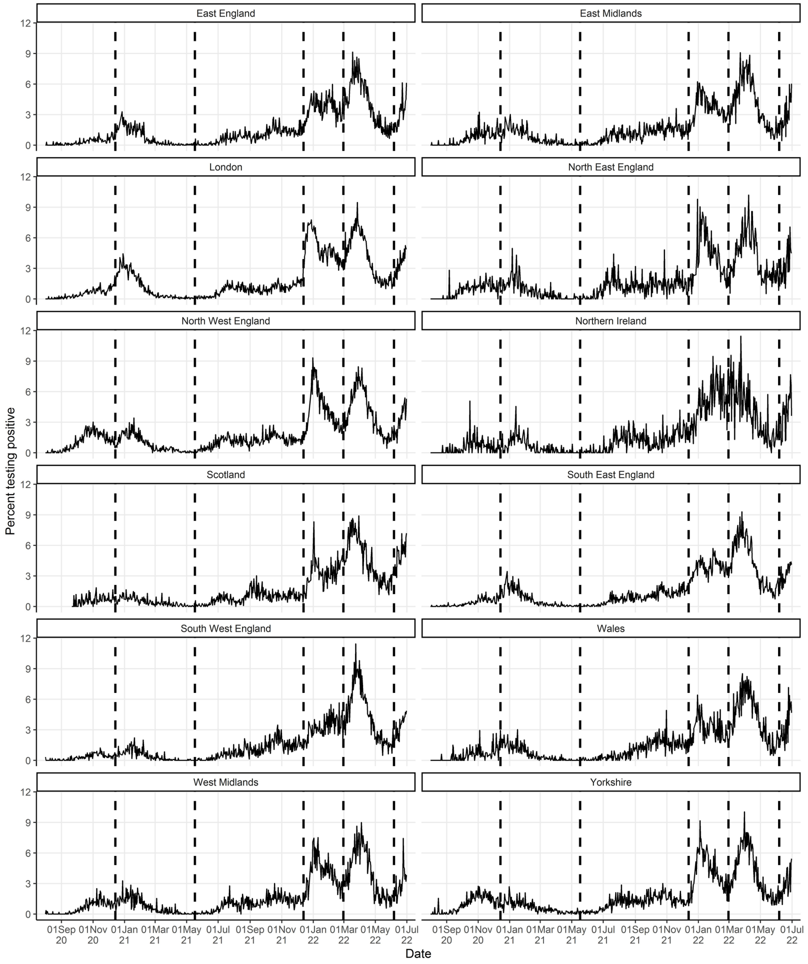

Note: Vertical dashed lines indicate periods when new variants became dominant, defined as >50% of positive swabs with cycle threshold (Ct)<30 being S-gene target positive (ORF1ab+N+S, ORF1ab+S, N+S gene positivity) in the Covid-19 Infection Survey for the pre-Alpha period (01 August 2020 - 13 December 2020), the Delta variant (17 May 2021 – 12 December 2021), and the Omicron BA.2 variant (28 February 2022 – 5 June 2022), and >50% Ct<30 S-gene target negative (ORF1ab+N gene positivity) for the Alpha variant (14 December 2020 – 16 May 2021), Omicron BA.1 variant (13 December 2021 – 27 February 2022), and Omicron BA4/BA.5 (6 June 2022 onwards) .

Figure S5: Predicted percentage of visits testing positive for SARS-CoV-2 from ISR (blue) and GAMs (orange) for all regions

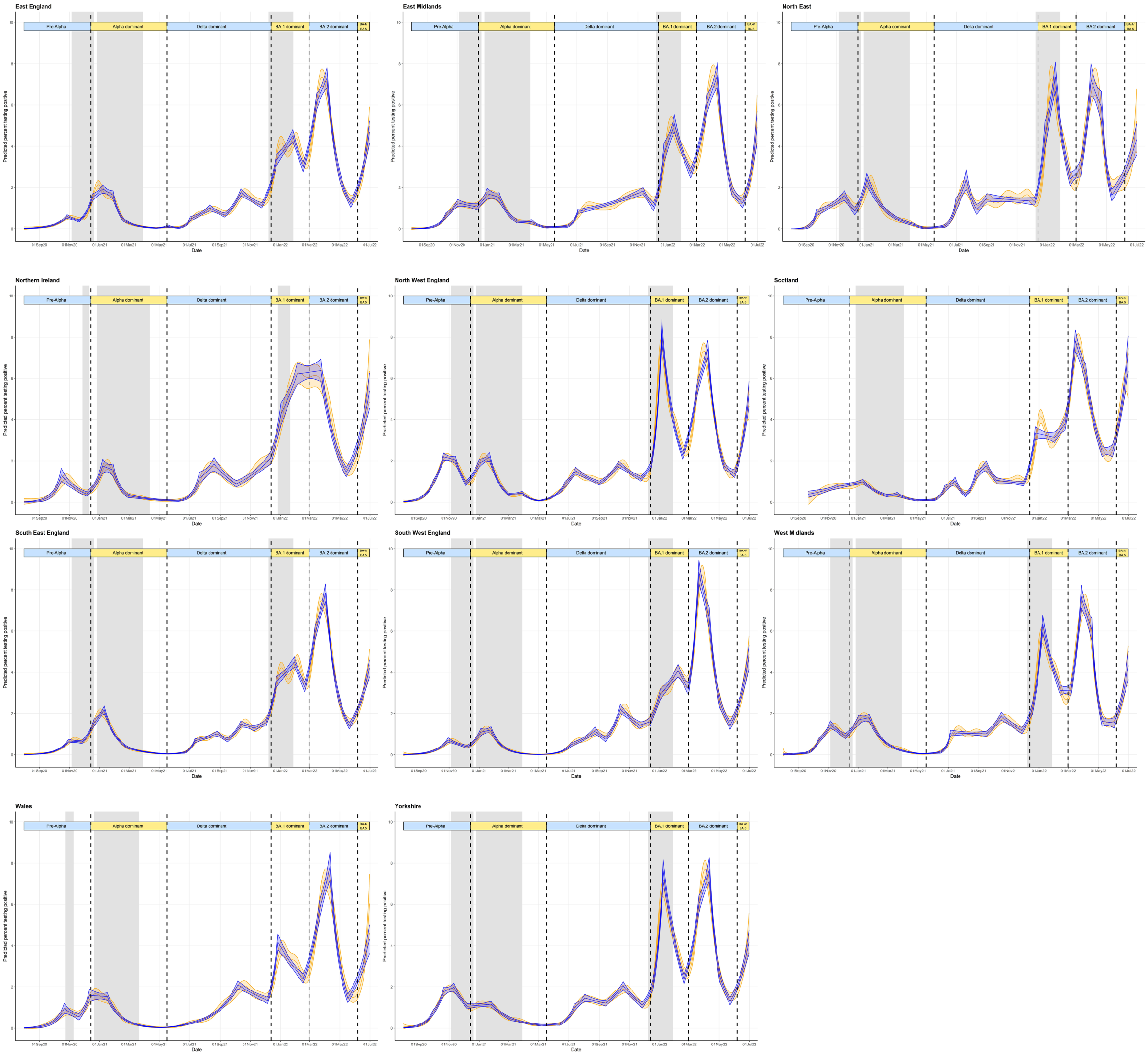

Note: Vertical dashed lines indicate periods when new variants became dominant, defined as >50% of positive swabs with cycle threshold (Ct)<30 being S-gene target positive (ORF1ab+N+S, ORF1ab+S, N+S gene positivity) in the Covid-19 Infection Survey for the pre-Alpha period (01 August 2020 - 13 December 2020), the Delta variant (17 May 2021 – 12 December 2021), and the Omicron BA.2 variant (28 February 2022 – 5 June 2022), and >50% Ct<30 S-gene target negative (ORF1ab+N gene positivity) for the Alpha variant (14 December 2020 – 16 May 2021), Omicron BA.1 variant (13 December 2021 – 27 February 2022), and Omicron BA4/BA.5 (6 June 2022 onwards) . Gray shaded indicate periods where stay/work from home laws were enforced, although specific restrictions varied across the time series.

Figure S6: The relative percentage decrease (blue) or increase (red) in positivity on the date of the detected change-point compared with positivity 4 weeks later. Results are presented for second derivatives of generalised additive models (GAMs; top) and iterative sequential regression (ISR; bottom). Models run on all 12 regions across the full time-series are included.

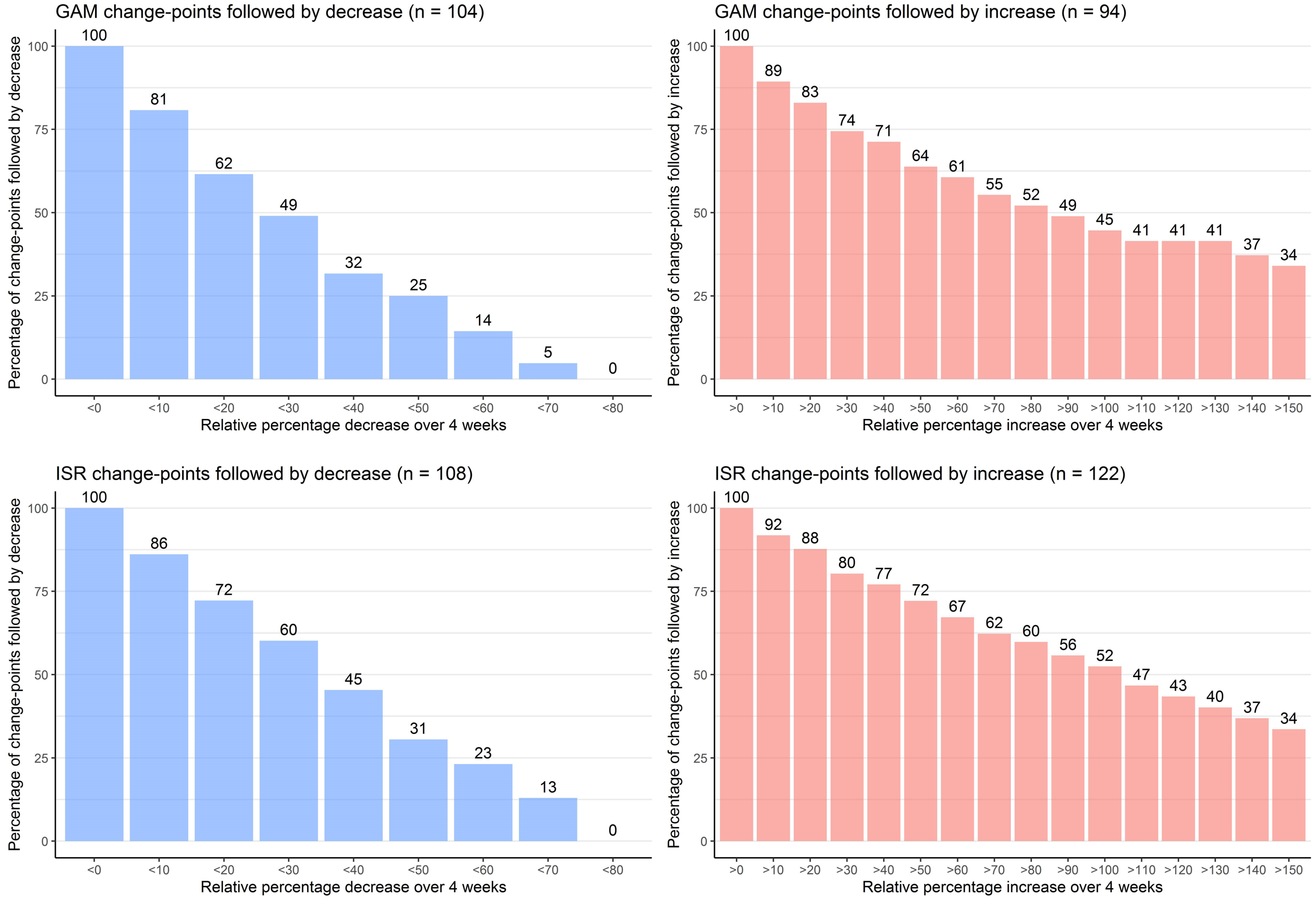

Figure S7: Change-points found by generalised additive models (GAMs), run successively over 32-week periods from September 2020-June 2022 summarised by the number of successive GAMs each change-point was confirmed by (zero to five), and whether the change-points were identified by iterative sequential regression (ISR; colour of circle) (top panel). Predicted positivity from final GAM for reference (bottom panel). Results are for Northern Ireland only.

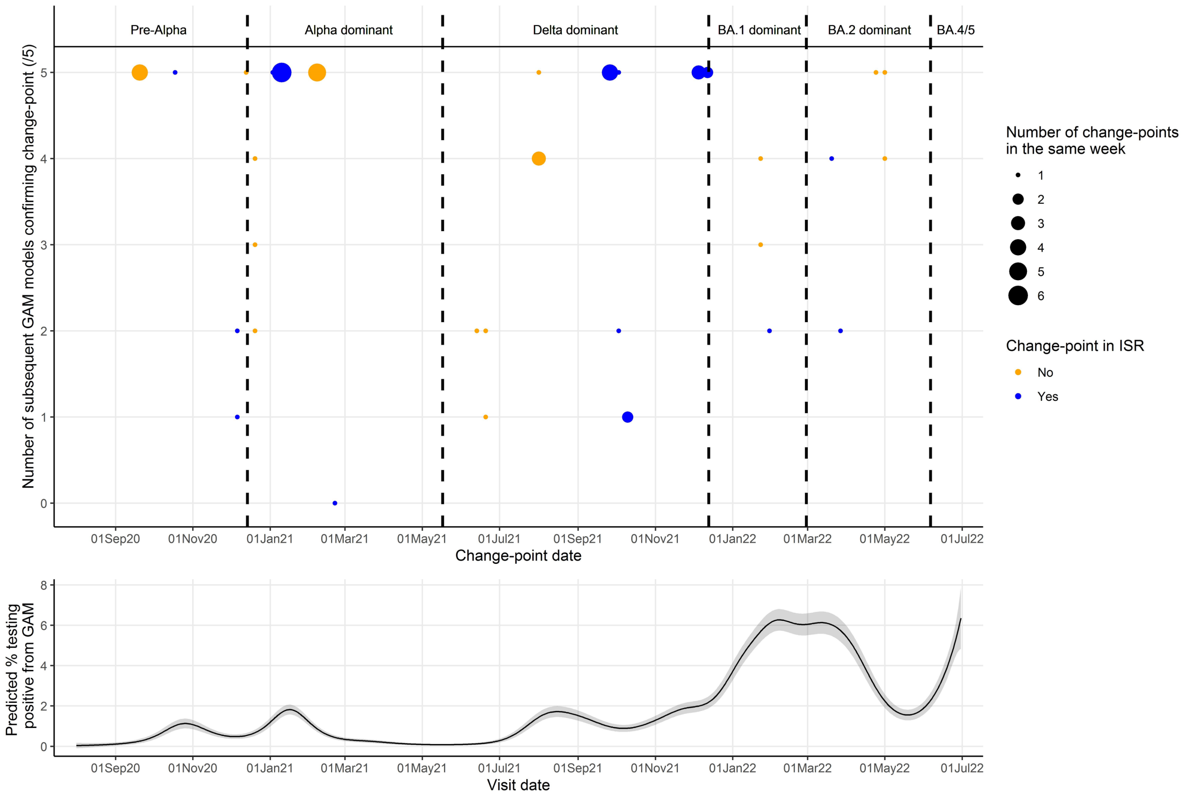

Note: Change-points in the same week (starting Monday) found in the same number of subsequent models were grouped together (indicated by size of circle). Points are blue if at least one change-point in that week was also found by ISR, and orange is no change-points in that week were found by ISR.

Figure S8: Predicted positivity (A), first derivatives (B), and second derivatives (C) estimated from GAMs fitted on the entire time-series for each geographical region, but only presented from 1st March to 30th June 2020. Original change-points based on the second derivative are shown in vertical blue lines, and additional change-points based on the first derivative are shown in vertical orange lines.

A: Predicted positivity

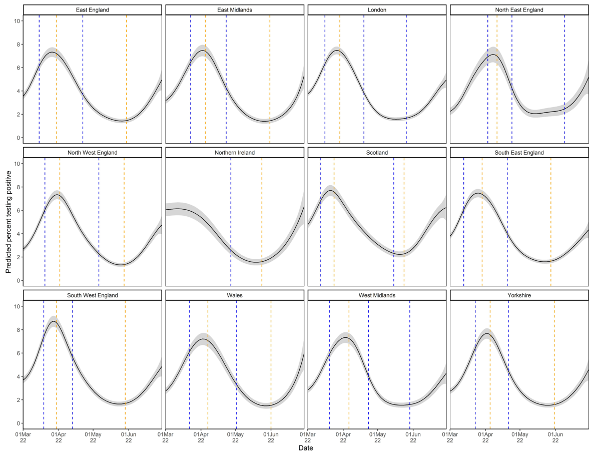

B: First derivative

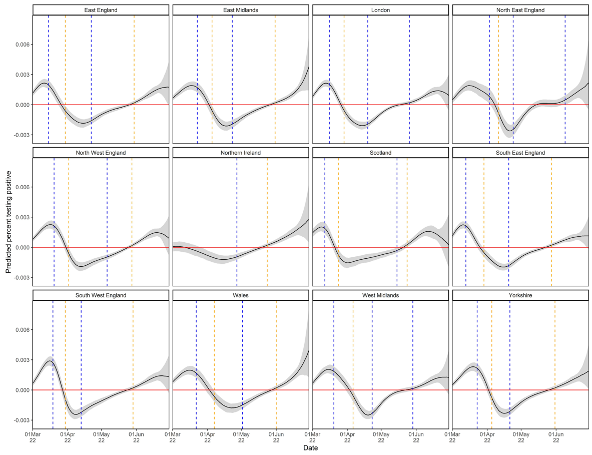

C: Second derivative

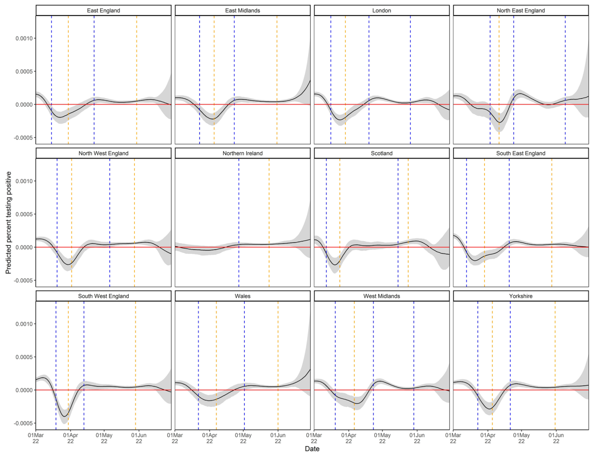

Figure S9: Predicted percentage testing positive for SARS-CoV-2 from ISR (blue) and GAMs (orange) for models run separately by age group for London

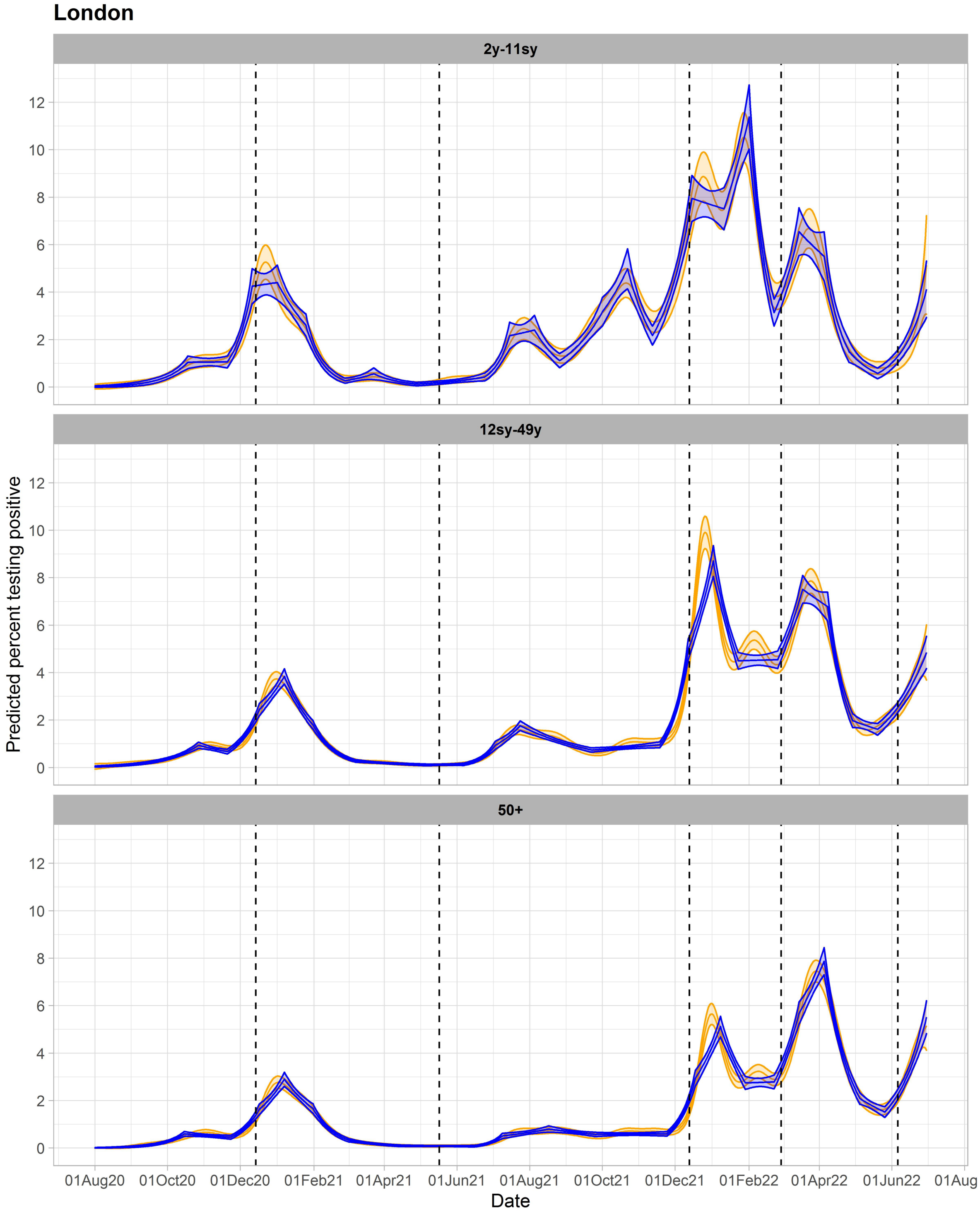

Note: Vertical dashed lines indicate periods when new variants became dominant, defined as >50% of positive swabs with cycle threshold (Ct)<30 being S-gene target positive (ORF1ab+N+S, ORF1ab+S, N+S gene positivity) in the Covid-19 Infection Survey for the pre-Alpha period (01 August 2020 - 13 December 2020), the Delta variant (17 May 2021 – 12 December 2021), and the Omicron BA.2 variant (28 February 2022 – 5 June 2022), and >50% Ct<30 S-gene target negative (ORF1ab+N gene positivity) for the Alpha variant (14 December 2020 – 16 May 2021), Omicron BA.1 variant (13 December 2021 – 27 February 2022), and Omicron BA4/BA.5 (6 June 2022 onwards).

Figure S10: Predicted percentage testing positive with first and second derivatives for different age groups in London.

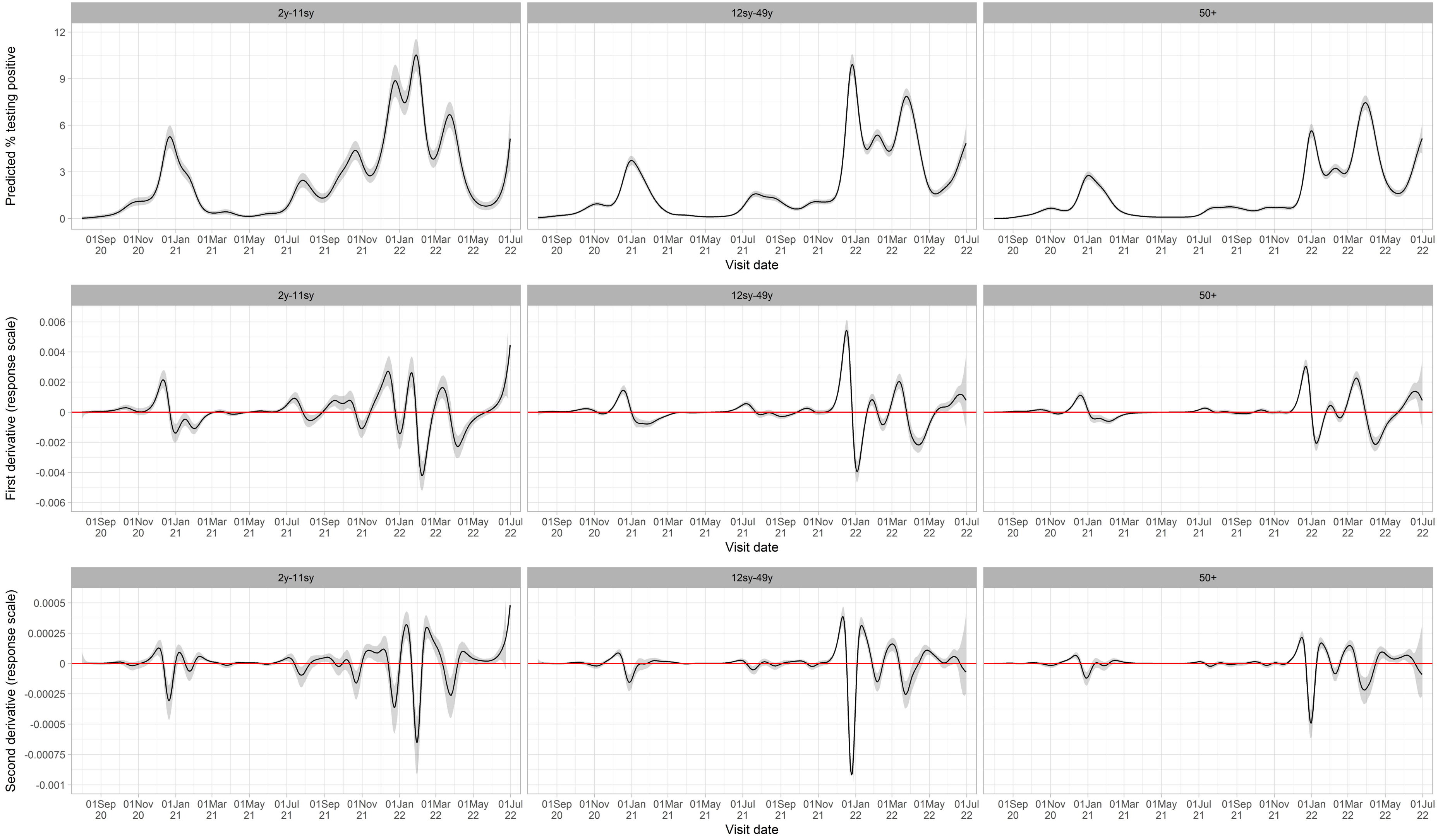

Figure S11: Raw daily percentage testing positive split by S-gene target positive and S-gene target failure.

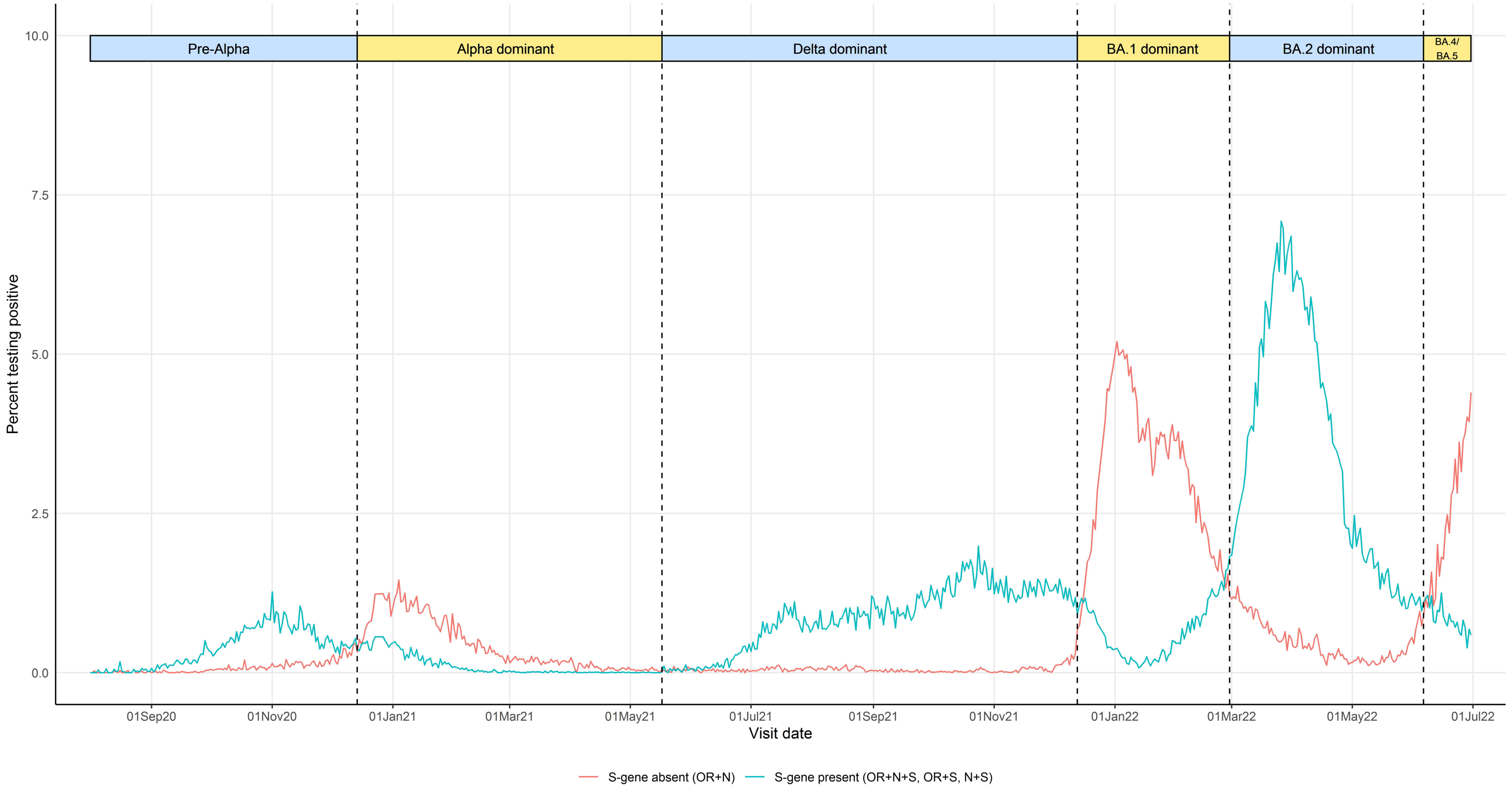

Note: Vertical dashed lines indicate periods when new variants became dominant, defined as >50% of positive swabs with cycle threshold (Ct)<30 being S-gene target positive (ORF1ab+N+S, ORF1ab+S, N+S gene positivity) in the Covid-19 Infection Survey for the pre-Alpha period (01 August 2020 - 13 December 2020), the Delta variant (17 May 2021 – 12 December 2021), and the Omicron BA.2 variant (28 February 2022 – 5 June 2022), and >50% Ct<30 S-gene target negative (ORF1ab+N gene positivity) for the Alpha variant (14 December 2020 – 16 May 2021), Omicron BA.1 variant (13 December 2021 – 27 February 2022), and Omicron BA4/BA.5 (6 June 2022 onwards).

Figure S12: Raw daily percentage testing positive split by S-gene target positive and S-gene target failure, by region.

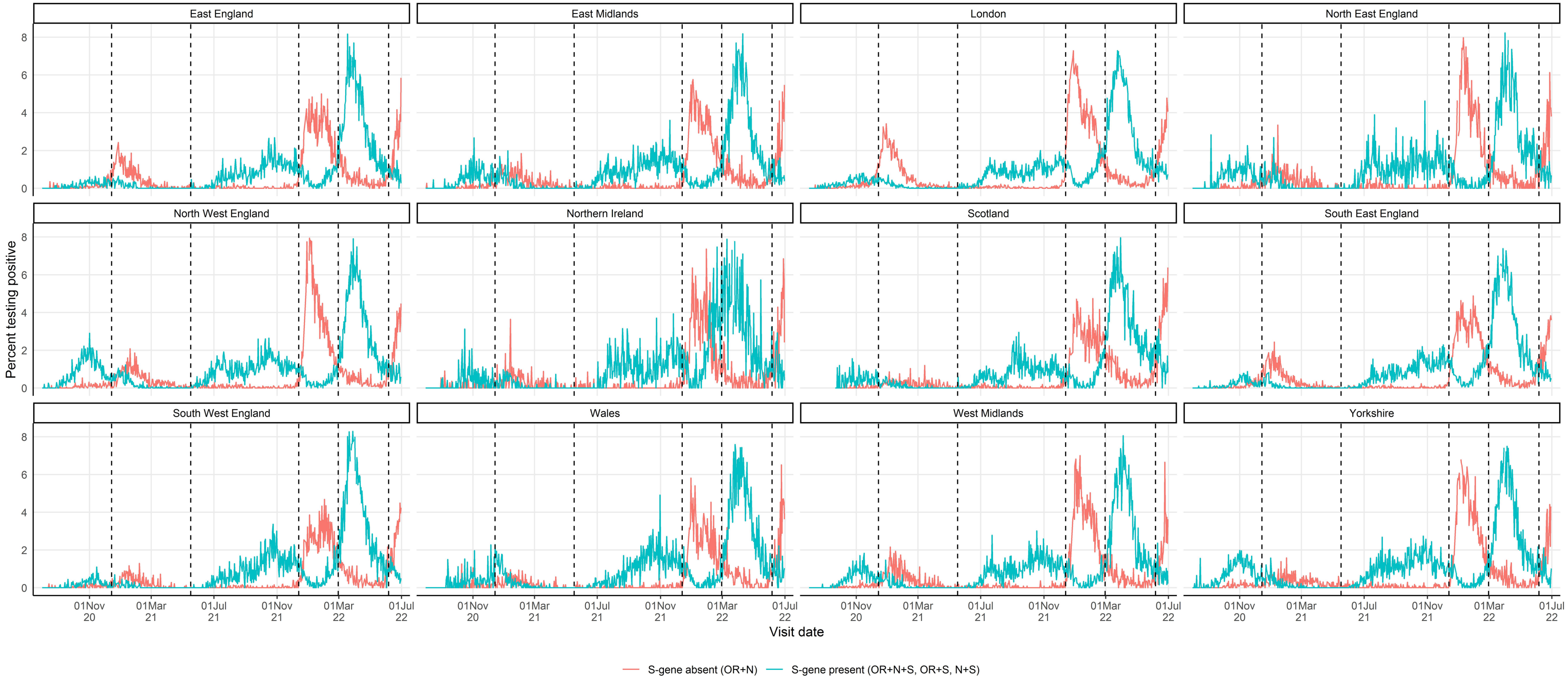

Note: Vertical dashed lines indicate periods when new variants became dominant, defined as >50% of positive swabs with cycle threshold (Ct)<30 being S-gene target positive (ORF1ab+N+S, ORF1ab+S, N+S gene positivity) in the Covid-19 Infection Survey for the pre-Alpha period (01 August 2020 - 13 December 2020), the Delta variant (17 May 2021 – 12 December 2021), and the Omicron BA.2 variant (28 February 2022 – 5 June 2022), and >50% Ct<30 S-gene target negative (ORF1ab+N gene positivity) for the Alpha variant (14 December 2020 – 16 May 2021), Omicron BA.1 variant (13 December 2021 – 27 February 2022), and Omicron BA4/BA.5 (6 June 2022 onwards).

Figure S13: Predicted daily percentage of visits testing positive for SARS-CoV-2 from ISR (blue) and GAMs (orange) for London only, split by SGTP and SGTF

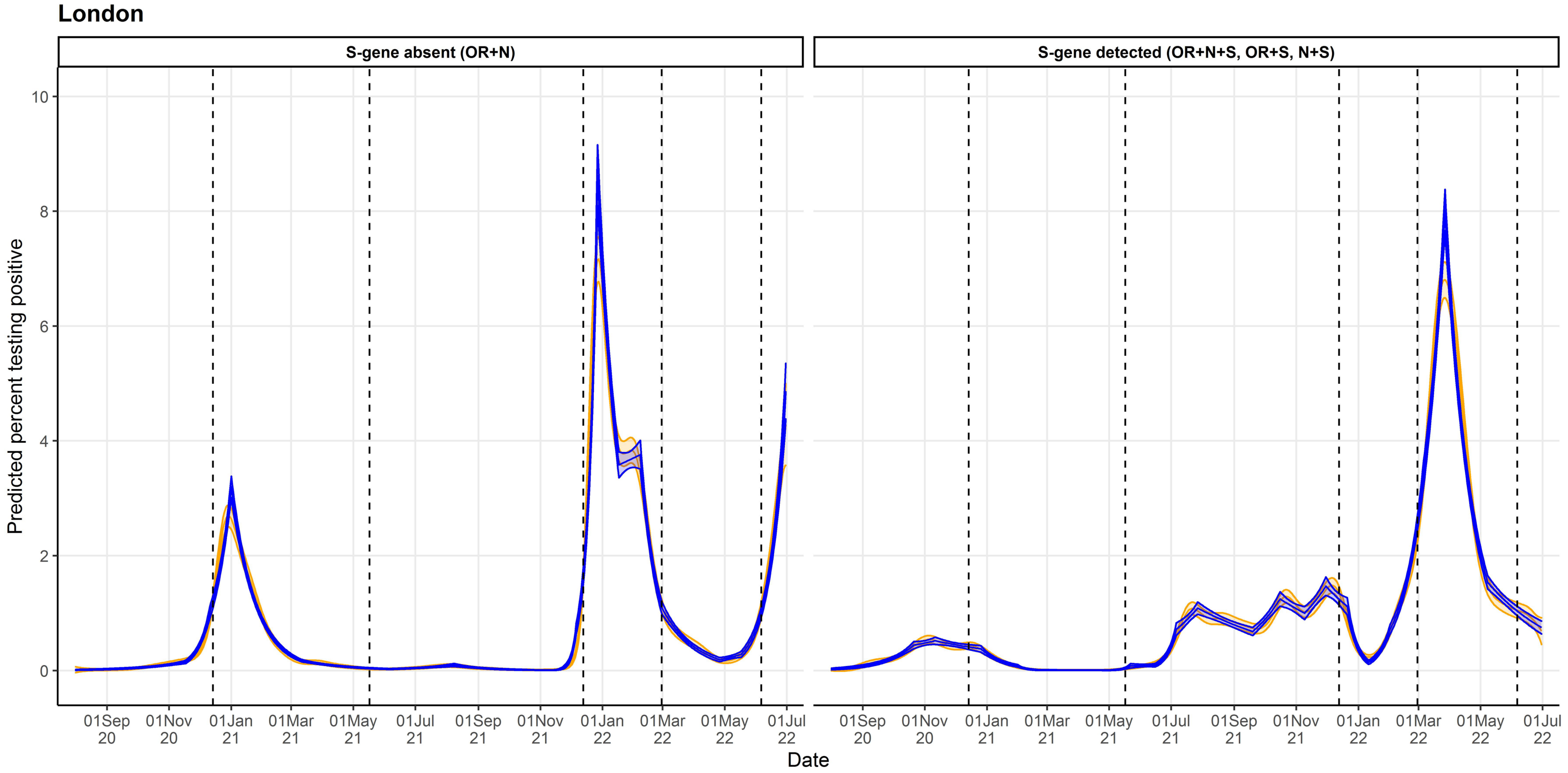

Note: Vertical dashed lines indicate periods when new variants became dominant, defined as >50% of positive swabs with cycle threshold (Ct)<30 being S-gene target positive (ORF1ab+N+S, ORF1ab+S, N+S gene positivity) in the Covid-19 Infection Survey for the pre-Alpha period (01 August 2020 - 13 December 2020), the Delta variant (17 May 2021 – 12 December 2021), and the Omicron BA.2 variant (28 February 2022 – 5 June 2022), and >50% Ct<30 S-gene target negative (ORF1ab+N gene positivity) for the Alpha variant (14 December 2020 – 16 May 2021), Omicron BA.1 variant (13 December 2021 – 27 February 2022), and Omicron BA4/BA.5 (6 June 2022 onwards).

Tables S1-S8

Table S1: Characteristics of all visits included in analysis, split by swab result

Characteristic	Positive, n (%) or median (IQR)	Negative, n (%) or median (IQR)	Total, n (%) or median (IQR)
**Age (years)**	47 (27, 62)	53 (34, 67)	53 (34, 67)
**Age group**			
2y-11sy	25,168 (17)	977,775 (11)	1,002,943 (11)
12sy-49y	54,950 (37)	2,868,355 (33)	2,923,305 (33)
50+	67,160 (45)	4,805,671 (55)	4,872,831 (55)
**Sex**			
Male	70,733 (48)	4,035,518 (46)	4,106,251 (46)
Female	76,545 (51)	4,616,283 (53)	4,692,828 (53)
**Geographical region**			
Scotland	10,679 (7)	648,775 (7)	659,454 (7)
North West England	18,801 (12)	994,441 (11)	1,013,242 (11)
North East England	5,831 (3)	321,657 (3)	327,488 (3)
Yorkshire	12,889 (8)	719,130 (8)	732,019 (8)
East Midlands	9,089 (6)	547,238 (6)	556,327 (6)
West Midlands	11,042 (7)	658,057 (7)	669,099 (7)
East England	12,834 (8)	829,889 (9)	842,723 (9)
Wales	6,725 (4)	428,267 (4)	434,992 (4)
London	26,286 (17)	1,443,346 (16)	1,469,632 (16)
South East England	17,758 (12)	1,128,635 (13)	1,146,393 (13)
South West England	10,823 (7)	686,459 (7)	697,282 (7)
Northern Ireland	4,521 (3)	245,907 (2)	250,428 (2)

Table S2: Characteristics of SARS-CoV-2 positive swabs, split by period in which different variants dominated

Characteristic	Pre-alpha	Alpha	Delta	Omicron BA.1	Omicron BA.2	Omicron BA.4/BA.5
**Date range**	01 August 2020 to 13 December 2020	14 December 2020 to 16 May 2021	17 May 2021 to 12 December 2021	13 December 2021 to 27 February 2022	28 February 2022 to 05 June 2022	06 June 2022, 30 June 2022
**Number of positives, n (%)**	12,263 (8)	16,667 (11)	26,805 (18)	39,620 (27)	45,318 (31)	6,582 (4)
**Ct value, median (IQR)**	28 (21, 32)	30 (22, 33)	25 (19, 31)	24 (19, 30)	24 (20, 30)	23 (19, 28)
**Ct < 30, n (%)**	7,203 (59)	8,307 (50)	19,107 (71)	30,432 (77)	34,200 (75)	5,402 (82)
**SGTF (% all pos) [% Ct <30]**	705 (6) [10]	6,593 (40) [79]	242 (1) [1]	25,462 (64) [84]	3,206 (7) [9]	4,054 (62) [75]
**S-gene detected (% all pos) [% Ct <30]**	6,452 (53) [90]	1,669 (10) [20]	18,844 (70) [99]	4,925 (12) [16]	30,985 (68) [91]	1,345 (20) [25]
**Ct ≥ 30 (% all pos)**	5,106 (42)	8,405 (50)	7,719 (29)	9,233 (23)	11,127 (25)	1,183 (18)

Note: Excluding 23 positives results without Ct values or genes detected available. Epochs were defined by >50% of positive swabs with cycle threshold (Ct)<30 being S-gene target positive (ORF1ab+N+S, ORF1ab+S, N+S gene positivity) in the Covid-19 Infection Survey for the pre-Alpha period (01 August 2020 - 13 December 2020), the Delta variant (17 May 2021 – 12 December 2021), and the Omicron BA.2 variant (28 February 2022 – 5 June 2022), and >50% Ct<30 S-gene target negative (ORF1ab+N gene positivity) for the Alpha variant (14 December 2020 – 16 May 2021), Omicron BA.1 variant (13 December 2021 – 27 February 2022), and Omicron BA4/BA.5 (6 June 2022 onwards) .

Table S3: Change-points corresponding to periods corresponding to emergence of four key SARS-CoV-2 variants found by iterative sequential regression (ISR) and second derivatives of generalised additive models (GAM) for each geographical region, run on the full time-series, as shown in Figure 2.

Region	Coincident Variant	GAM breakpoint (DD.MM.YYYY)	ISR breakpoint (DD.MM.YYYY)	ISR detection date	Days between ISR and GAM change-point*	Days between ISR change-point and detection
**East England**	Alpha	20.11.2020	20.11.2020	14.12.2020	0	24
	Delta	05.06.2021	09.06.2021	06.07.2021	-4	27
	BA.1	27.11.2021	24.11.2021	18.12.2021	3	24
	BA.2	14.02.2022	16.02.2022	12.03.2022	-2	24
**East Midlands**	Alpha	**Not found**	11.12.2020	04.01.2021	n/a	24
	Delta	01.06.2021	12.06.2021	06.07.2021	-11	24
	BA.1	28.11.2021	03.12.2021	27.12.2021	-5	24
	BA.2	15.02.2022	16.02.2022	12.03.2022	-1	24
**London**	Alpha	20.11.2020	26.11.2020	20.12.2020	-6	24
	Delta	09.06.2021	06.06.2021	30.06.2021	3	24
	BA.1	24.11.2021	30.11.2021	24.12.2021	-6	24
	BA.2	15.02.2022	28.02.2022	24.03.2022	-13	24
**North East**	Alpha	06.12.2020	08.12.2020	01.01.2021	-2	24
	Delta	25.05.2021	06.06.2021	09.07.2021	-12	33
	BA.1	04.12.2021	06.12.2021	30.12.2021	-2	24
	BA.2	15.02.2022	16.02.2022	12.03.2022	-1	24
**Northern Ireland**	Alpha	19.11.2020	05.12.2020	04.01.2021	-16	30
	Delta	09.06.2021	09.06.2021	03.07.2021	0	24
	BA.1	07.12.2021	12.12.2021	05.01.2022	-5	24
	BA.2	n/a	n/a	n/a	n/a	n/a
**North West**	Alpha	01.12.2020	05.12.2020	29.12.2020	-4	24
	Delta	14.04.2021	01.05.2021	25.05.2021	-17	24
	BA.1	30.11.2021	24.11.2021	18.12.2021	6	24
	BA.2	12.02.2022	16.02.2022	12.03.2022	-4	24
**Scotland**	Alpha	**Not found**	20.11.2020	23.12.2020	n/a	33
	Delta	26.05.2021	03.06.2021	27.06.2021	-8	24
	BA.1	01.12.2021	30.11.2021	24.12.2021	1	24
	BA.2	15.01.2022	01.02.2022	25.02.2022	-17	24
**South East**	Alpha	23.11.2020	26.11.2020	20.12.2020	-3	24
	Delta	08.06.2021	21.06.2021	15.07.2021	-13	24
	BA.1	05.12.2021	03.12.2021	27.12.2021	2	24
	BA.2	15.02.2022	19.02.2022	15.03.2022	-4	24
**South West**	Alpha	26.11.2020	08.12.2020	01.01.2021	-12	24
	Delta	23.05.2021	01.05.2021	25.05.2021	22	24
	BA.1	06.11.2021	21.11.2021	18.12.2021	-15	27
	BA.2	19.02.2022	28.02.2022	24.03.2022	-9	24
**Wales**	Alpha	14.11.2020	20.11.2020	14.12.2020	-6	24
	Delta	01.06.2021	27.06.2021	01.10.2021	-26	96
	BA.1	01.12.2021	06.12.2021	30.12.2021	-5	24
	BA.2	14.02.2022	16.02.2022	12.03.2022	-2	24
**West Midlands**	Alpha	30.11.2020	05.12.2020	29.12.2020	-5	24
	Delta	25.05.2021	10.05.2021	06.06.2021	15	27
	BA.1	28.11.2021	27.11.2021	21.12.2021	1	24
	BA.2	15.02.2022	13.02.2022	09.03.2022	2	24
**Yorkshire**	Alpha	03.12.2020	08.12.2020	01.01.2021	-5	24
	Delta	09.06.2021	12.06.2021	06.07.2021	-3	24
	BA.1	27.11.2021	27.11.2021	21.12.2021	0	24
	BA.2	14.02.2022	19.02.2022	15.03.2022	-5	24

*Negative values indicate earlier occurrence of change-points using GAMs, compared with ISR.

Table S4: Comparison of change-points detected by generalised additive models run on the full time series from 1st August 2020, 16-week, 24-week, and 32-week periods for London

Model end date (days from 1st August 2020)	Change-point dates from model run from 1st August 2020 [duration*, days]	Change-point dates (days between current change-point and change-point in full model) [duration*, days]		
		16-week model (112 days)	24-week model (168 days)	32-week model (224 days)
26.11.2020 (118 days)	06.11.2020 [14]	04.11.2020 (-2) [17]	n/a	n/a
21.01.2021 (174 days)	09.12.2020 [5]	-	11.12.2020 (2) [3]	n/a
	23.12.2020 [9]	25.12.2020 (2) [6]	24.12.2020 (1) [7]	n/a
	-	05.01.2021 (n/a) [3]	-	n/a
18.03.2021 (230 days)	10.02.2021 [1]	-	-	10.02.2021 (0) [3]
	12.02.2021 [2]	-	-	14.02.2021 (-2) [1]
	-	-	-	21.02.2021 (n/a) [2]
	23.02.2021 [9]	26.02.2021 (3) [7]	-	24.02.2021 (-1) [9]
13.05.2021 (286 days)	No change-points	No change-points	No change-points	No change-points
08.07.2021 (342 days)	13.06.2021 [17]	-	-	14.06.2021 (-1) [19]
02.09.2021 (398 days)	13.07.2021 [12]	14.07.2021 (1) [9]	14.07.2021 (1) [11]	14.07.2021 (1) [12]
28.10.2021 (454 days)	-	10.09.2021 (n/a) [45]	-	-
	04.10.2021 [6]	-	-	04.10.2021 (0) [5]
23.12.2021 (510 days)	04.11.2021 [10]	-	-	-
	27.11.2021 [27]	04.12.2021 (7) [12]	03.12.2021 (6) [14]	02.12.2021 (5) [16]
17.02.2022 (566 days)	06.01.2022 [18]	12.01.2022 (6) [6]	12.01.2022 (6) [5]	11.01.2022 (5) [8]
	02.02.2022 [5]	-	-	-

*Duration = number of days which the credible interval of the second derivative did not contain zero

Note: Change-points recorded as "n/a" are not applicable as the 24-week and/or 32-week model is identical to the model run from 1st August 2020.

Table S5: Comparison of change-points found in GAMs run successively on 32-week periods with the GAMs run on the full time-series, for London only. Comparisons include change-points found in the full GAM and whether they were found in the 32-week models (A) and dates of change-points found in the last 4-weeks of the 32-week models but not within +/-7 days of a change-point in the full GAM (B)

A: Change-points found in the full GAM and whether they were found in the 32-week models

Change-point in full GAM	Found in last 4-weeks of 32-week GAM	Date of change-point(s) identified in last 4 weeks of 32-week GAM	Last date included in 32-week model which change-point was found in	Days between change-point and last date included in model
09.06.2021	Yes	12.06.2021	01.07.2021	19
12.07.2021	Yes	14.07.2021,<br>16.07.2021	05.08.2021,<br>29.07.2021	22,<br>13
25.09.2021	Yes	02.10.2021	21.10.2021	19
15.10.2021	Yes	18.10.2021,<br>19.10.2021	11.11.2021,<br>04.11.2021	24,<br>16
01.11.2021	Yes	07.11.2021	25.11.2021	18
24.11.2021	No	-	-	-
20.12.2021	Yes	19.12.2021,<br>19.12.2021,<br>20.12.2021	06.01.2022,<br>13.01.2022,<br>30.12.2021	18,<br>25,<br>10
06.01.2022	Yes	11.01.2022,<br>11.01.2022	27.01.2022,<br>03.02.2022	16,<br>23
29.01.2022	No	-	-	-
15.02.2022	No	-		-
16.03.2022	No	-		-
19.04.2022	Yes	24.04.2022	12.05.2022	18
26.05.2022	No	-	-	-

B: Dates of change-points found in the last 4-weeks of the successive 32-week models but not within ±7 days of a change-point in the full GAM

Date of change-point identified in successive 32-week GAM but not in the full time-series GAM	Last date included in the successive 32-week model in which the change-point was found	Days between change-point and last date in model
22.02.2021	18.03.2021	24
03.10.2021	28.10.2021	25
09.11.2021	01.12.2021	23
02.12.2021	16.12.2021	14
02.12.2021	23.12.2021	21
24.02.2022	10.03.2022	14
24.02.2022	17.03.2022	21
24.03.2022	07.04.2022	14
24.03.2022	14.04.2022	21

Table S6: Detection dates for GAMs and ISR for London and Northern Ireland

Geographical region	Change-point in final GAM	GAM detection date	ISR change-point	ISR detection date	Difference in GAM change-point & detection	Difference in ISR change-point & detection	Difference in GAM & ISR change-points	Difference in GAM & ISR detection
London	26.09.2020	22.10.2020	n/a	n/a	26	n/a	n/a	n/a
	n/a	n/a	15.10.2020	08.11.2020	n/a	24	n/a	n/a
	02.11.2020	19.11.2020	05.11.2020	29.11.2020	17	24	-3	-10
	20.11.2020	03.12.2020	26.11.2020	20.12.2020	13	24	-6	-17
	19.12.2020	07.01.2021	17.12.2020	10.01.2021	19	24	2	-3
	n/a	n/a	07.01.2021	31.01.2021	n/a	24	n/a	n/a
	23.01.2021	n/a	28.01.2021	21.02.2021	n/a	24	-5	n/a
	05.02.2021	25.02.2021	n/a	n/a	20	n/a	n/a	n/a
	n/a	n/a	02.03.2021	26.03.2021	n/a	24	n/a	n/a
	n/a	n/a	01.05.2021	25.05.2021	n/a	24	n/a	n/a
	09.06.2021	01.07.2021	06.06.2021	30.06.2021	22	24	3	1
	12.07.2021	29.07.2021	06.07.2021	30.07.2021	17	24	6	-1
	n/a	n/a	27.07.2021	20.08.2021	n/a	24	n/a	n/a
	25.09.2021	21.10.2021	19.09.2021	13.10.2021	26	24	6	8
	15.10.2021	04.11.2021	16.10.2021	09.11.2021	20	24	-1	-5
	01.11.2021	25.11.2021	n/a	n/a	24	n/a	n/a	n/a
	n/a	n/a	09.11.2021	03.12.2021	n/a	24	NA	NA
	24.11.2021	n/a	30.11.2021	24.12.2021	NA	24	-6	NA
	20.12.2021	30.12.2021	21.12.2021	14.01.2022	10	24	-1	-15
	06.01.2022	27.01.2022	11.01.2022	04.02.2022	21	24	-5	-8
	29.01.2022	n/a	n/a	n/a	n/a	n/a	n/a	n/a
	n/a	n/a	07/02.2022	03.03.2022	n/a	24	n/a	n/a
	15.02.2022	23.06.2022	n/a	n/a	128	n/a	n/a	n/a
	n/a	n/a	28.02.2022	24.03.2022	n/a	24	n/a	n/a
	16.03.2022	19.05.2022	21.03.2022	14.04.2022	64	24	-5	35
	n/a	n/a	11.04.2022	05.05.2022	n/a	24	n/a	n/a
	19.04.2022	12.05.2022	n/a	n/a	23	NA	n/a	n/a
	n/a	n/a	02.05.2022	26.05.2022	n/a	24	n/a	n/a
	26.05.2022	n/a	23.05.2022	16.06.2022	n/a	24	3	n/a
Northern Ireland	14.09.2020	31.12.2020	n/a	n/a	108	n/a	n/a	n/a
	17.10.2020	10.12.2020	15.10.2020	08.11.2020	54	24	2	32
	19.11.2020	n/a	n/a	n/a	n/a	n/a	n/a	n/a
	n/a	n/a	05.12.2020	04.01.2021	n/a	30	n/a	n/a
	07.01.2021	21.01.2021	07.01.2021	31.01.2021	14	24	0	-10
	04.02.2021	11.03.2021	28.01.2021	21.02.2021	35	24	7	18
	n/a	n/a	27.02.2021	23.03.2021	n/a	24	n/a	n/a
	09.06.2021	12.08.2021	09.06.2021	03.07.2021	64	24	n/a	40
	n/a	n/a	21.07.2021	14.08.2021	n/a	24	n/a	n/a
	02.08.2021	26.08.2021	n/a	n/a	24	n/a	n/a	n/a
	n/a	n/a	20.08.2021	01.10.2021	n/a	42	n/a	n/a
	28.09.2021	28.10.2021	04.10.2021	28.10.2021	30	24	-6	0
	07.12.2021	06.01.2022	12.12.2021	05.01.2022	30	24	-5	1
	n/a	n/a	02.01.2022	13.02.2022	n/a	42	n/a	n/a
	n/a	n/a	04.02.2022	28.02.2022	n/a	24	n/a	n/a
	n/a	n/a	24.03.2022	17.04.2022	n/a	24	n/a	n/a
	27.04.2022	05.05.2022	n/a	n/a	8	n/a	n/a	n/a
	n/a	n/a	14.05.2022	07.06.2022	n/a	24	n/a	n/a

Table S7: Change-points from GAMs and ISR fitted on the full time-series for BA.4/ BA.5. Change-points from GAMs are presented for both change-points defined by the second derivative alone, as well as additional change-points based on the first derivative.

Geographical region	GAM change-point estimated using second derivative only	GAM change-point incorporating additional change-points from first derivative	ISR change-point
**East England**	Not found	30.05.2022	23.05.2022
**East Midlands**	Not found	31.05.2022	01.06.2022
**London**	26.05.2022	No change	23.05.2022
**North East**	09.06.2022	No change	11.05.2022
**Northern Ireland**	Not found	24.05.2022	14.05.2022
**North West**	Not found	28.05.2022	01.06.2022
**Scotland**	Not found	24.05.2022	29.05.2022
**South East**	Not found	28.05.2022	20.05.2022
**South West**	Not found	29.05.2022	23.05.2022
**Wales**	Not found	01.06.2022	17.05.2022
**West Midlands**	29.05.2022	No change	01.06.2022
**Yorkshire**	Not found	31.05.2022	23.05.2022

Table S8: Change-points from GAMs (run on the full time-series) and ISR run separately by age, separately by S gene detection and overall in London.

Variant	Sub-Group	GAM change-point date	ISR change-point date	ISR detection date	Days between GAM and ISR change-points*	Days between ISR change-point and detection date
**Alpha**	2y-11sy	19.11.2020	20.11.2020	14.12.2020	-1	24
	12sy-49	20.11.2020	20.11.2020	14.12.2020	0	24
	50+	20.11.2020	23.11.2020	17.12.2020	-3	24
	S-gene absent	19.11.2020	17.11.2020	11.12.2020	2	24
	**Overall model**	**20.11.2020**	**26.11.2020**	20.12.2020	-6	24
**Delta**	2y-11sy	16.06.2021	24.06.2021	21.07.2021	-8	27
	12sy-49	05.06.2021	06.06.2021	30.06.2021	-1	24
	50+	15.06.2021	15.06.2021	09.07.2021	0	24
	S-gene detected	10.06.2021	15.06.2021	09.07.2021	-5	24
	**Overall model**	**09.06.2021**	**06.06.2021**	30.06.2021	3	24
**Omicron BA.1**	2y-11sy	05.11.2021	12.11.2021	06.12.2021	-7	24
	12sy-49	19.11.2021	18.11.2021	12.12.2021	1	24
	50+	25.11.2021	24.11.2021	18.12.2021	1	24
	S-gene absent	11.11.2021	12.11.2021	09.12.2021	-1	27
	**Overall model**	**24.11.2021**	**30.11.2021**	24.12.2021	-6	24
**Omicron BA.2**	2y-11sy	09.02.2022	22.02.2022	18.03.2022	-13	24
	12sy-49y	18.02.2022	25.02.2022	21.03.2022	-7	24
	50y+	19.02.2022	22.02.2022	18.03.2022	-3	24
	S-gene detected	17.02.2022	11.01.2022	04.02.2022	37	24
	**Overall model**	**15.02.2022**	**28.02.2022**	24.03.2022	-13	24

Note:. Overall estimates from GAMs and ISR include all age groups with the outcome of all positives. *Negative values indicate earlier occurrence of change-points using GAMs, compared with ISR

REFERENCE LIST

1. Simpson GL. Modelling palaeoecological time series using generalised additive models. *Frontiers in Ecology and Evolution*. 2018:149.
2. Wood SN. Fast stable restricted maximum likelihood and marginal likelihood estimation of semiparametric generalized linear models. *Journal of the Royal Statistical Society: Series B (Statistical Methodology)*. 2011;73(1):3-36.
3. Wood SN. *Generalized additive models: an introduction with R*. chapman and hall/CRC; 2006.
